# Synthesis and Cytotoxicity Evaluation of Naphthalimide Derived *N*-Mustards

**DOI:** 10.3390/molecules19078803

**Published:** 2014-06-25

**Authors:** Qinghua Lou, Liyan Ji, Wenhe Zhong, Shasha Li, Siwang Yu, Zhongjun Li, Xiangbao Meng

**Affiliations:** State Key Laboratory of Natural and Biomimetic Drugs, Department of Chemical Biology, School of Pharmaceutical Sciences, Peking University, Beijing 100191, China; E-Mails: louqh@bjmu.edu.cn (Q.L.); jiliyan@bjmu.edu.cn (L.J.); vicentzhongwh@126.com (W.Z.); shashali@umich.edu (S.L.); zjli@bjmu.edu.cn (Z.L.)

**Keywords:** cytotoxicity, DNA alkylating agent, DNA intercalator, synthesis, *N*-mustard, naphthalimide

## Abstract

A series of *N*-mustards, which was conjugated to mono- or bis-naphthalimides with a flexible amine link, were synthesized and evaluated for cytotoxicity against five cancer cell lines (HCT-116, PC-3, U87 MG, Hep G2 and SK-OV-3). Several compounds displayed better activities than the control compound amonafide. Further evaluations by fluorescence spectroscopy studies and DNA-interstrand cross-linking assays revealed that the derivatives showed both alkylating and intercalating properties. Among the derivatives, the bis-naphthalimide *N*-mustard derivative **11b** was found to exhibit the highest cytotoxic activity and DNA cross-linking ability. Both **11b** and **7b** induce HCT-116 cell apoptosis by S phase arrest.

## 1. Introduction

**Figure 1:**
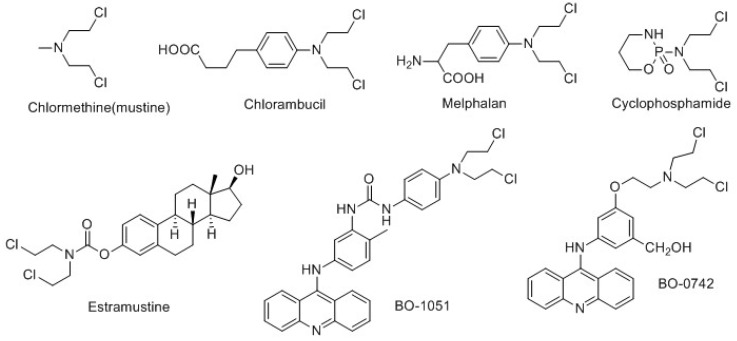
Chemical structures of *N*-mustard antineoplastic drugs and derivatives.

As the earliest agents used for chemotherapy, DNA alkylating nitrogen mustards have been widely utilized in oncology treatment, and many such agents are still in clinical use (Figure 1) [1,2,3,4,5]. However, progress in developing new *N*-mustard agents is limited due to its drawbacks [6]. Its necessarily high chemical reactivity leads to serious adverse effects [3,7,8] by randomly alkylating other cellular nucleophiles. They lack specific affinity to tumor cells and induce bone marrow toxicity [6,9]. To overcome those drawbacks, one of the strategies has been to synthesize bifunctional compounds by linking *N*-mustards with DNA-affine molecules, such as DNA-intercalators (e.g., acridines [6,9,10], cyclopentanthraquinone [11]) or DNA minor groove binders (e.g., distamycin A and related analogues [12,13,14]). Previous research has demonstrated that linking with an appropriate carrier can modify the specificity of DNA alkylation and thus improve the therapeutic efficacy of *N*-mustard agents. This strategy has been widely applied in the search for new drugs [9,15,16].

**Figure 2:**
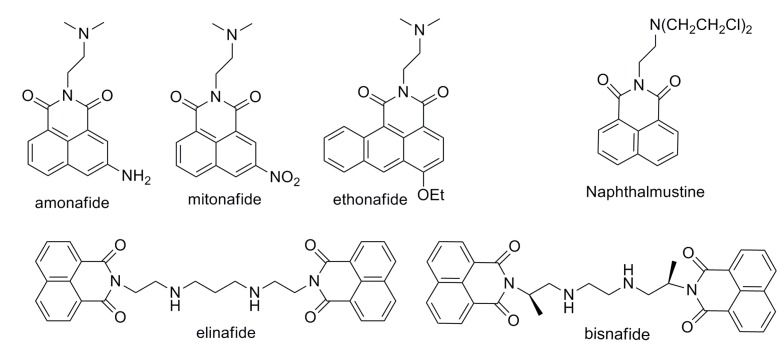
Structures of naphthalimides and *N*-mustard analogs.

Naphthalimide, a well-defined DNA-intercalator, has been extensively investigated as an antitumor agent [17]. Several naphthalimide derivatives such as mitonafide, amonafide, ethonafide, elinafide, and bisnafide (Figure 2) are currently undergoing clinical trials, [17]. Elinafide and bisnafide, which are bis-intercalators prepared by linking two naphthalimide groups with a polyamine linker, showed much higher cytotoxicity than the mono-intercalators mitonafide and amonafide [18,19]. However, only limited research have been done in the area of linking *N*-mustard with DNA-binding naphthalimide, and they showed enhanced cytotoxicities [20,21,22,23].

Herein, we designed and synthesized *N*-mustard derivatives of naphthalimides by linking the *N*-mustard moiety with naphthalimides. The conjugates acted as both alkylating agents and intercalators. Encouraged by the generally stronger cytotoxicities and high binding capacities of the bis-intercalators, we decided to synthesize both mono and bis-naphthalimide [18] conjugates to compare their cytotoxic activities.

## 2. Results and Discussion

### 2.1. Chemistry

**Scheme 1:**
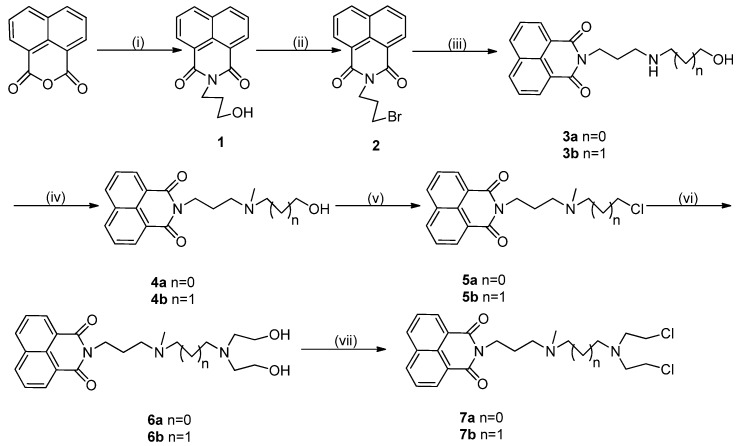
Synthesis of compounds **7a**–**b**.

To attach the *N*-mustard moiety to the naphathalimide core, we synthesized compound **2** from naphthalic anhydride [24,25]. Compound **2** was refluxed with an aminoalcohol in CH_3_CN to yield the key precursor compound **3**, which was converted to compound **4** by refluxing in HCHO and HCOOH. The chlorination of alcohol **4** in the presence of SOCl_2_ to produce **5** was successful [26]. The *N*-mustard precursor **6** was synthesized by condensing compound **5** with diethanolamine in the presence of K_2_CO_3_/KI [27]. The final target compound **7** was obtained from **6** by reacting with SOCl_2_ (Scheme 1).

*Reagents and conditions:* (i) 3-amino-1-propanol, EtOH, reflux; (ii) PPh_3_/NBS, CH_2_Cl_2_, rt; (iii) aminoalcohol, K_2_CO_3_, CH_3_CN, 70 °C; (iv) HCHO/HCOOH, reflux; (v) SOCl_2_, CHCl_3_, rt; (vi) diethanolamine, K_2_CO_3_/KI, CH_3_CN, reflux; (vii) SOCl_2_, reflux.

**Scheme 2:**
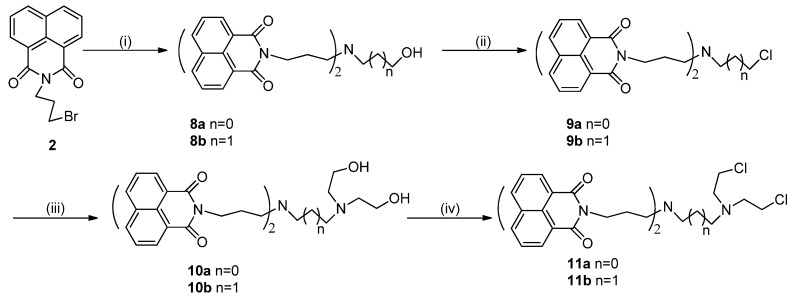
Synthesis of bis-naphthalimide derivatives.

The synthesis of bis-naphthalimide derivatives is illustrated in Scheme 2. Starting from compound **2**, the target compounds **11a**,**b** were obtained following the procedures described below.

*Reagents and conditions:* (i) aminoalcohol, K_2_CO_3_, CH_3_CN, 80 °C; (ii) SOCl_2_, CH_2_Cl_2_, rt; (iii) diethanolamine, K_2_CO_3_/KI, CH_3_CN, reflux; (iv) SOCl_2_, reflux.

### 2.2. Biological Assay

#### 2.2.1. Cytotoxic Activity

The *N*-mustard naphthalimide derivatives **7a**–**b**, **11a**–**b** and the precursors **6a**–**b**, **10a**–**b** in their dihydrochloride forms were screened for their cytotoxic activities against five cancer cell lines, which were HCT-116 (colorectal carcinoma), PC-3 (prostate carcinoma), U87 MG (brain tumor), Hep G2 (liver cancer), and SK-OV-3 (ovarian cancer), with an *in vitro* cell growth assay using amonafide as positive control. The results were listed in Table 1. The activity of compound **11b** was the highest among all the compounds and was increased by several fold (1.4–11) compared to amonafide, while compound **7b** showed a similar activity with amonafide. *N*-mustards **7b** and **11b** displayed more activity than their precursors **6b**, **10b**. Furthermore, compared with compounds **7b** and **11b**, **7a** and **11a** unexpectedly showed much lower activities (>10 μM). Because quaternary ammonium salts of **7a** and **11a** were used in this cytotoxicity assay, the six-membered rings in **7a** and **11a**, which make them less flexible, may be the reason for the lower activities.

**Table 1:** IC_50_ values of naphthalimide derivatives against five cancer cell lines ^a^.

Compound ^b^	IC_5_ (μM)				
	HCT-116	PC-3	U87MG	Hep G2	SK-OV-3
**6a**	73.77 ± 2.54	225.80 ± 6.41	40.80 ± 3.14	42.81 ± 26.39	63.35 ± 5.42
**6b**	6.96 ± 1.64	69.97 ± 10.14	10.24 ± 0.19	9.39 ± 4.37	12.76 ± 1.40
**7a**	33.29 ± 3.13	73.10 ± 14.61	10.77 ± 0.07	15.38 ± 2.88	35.31 ± 5.69
**7b**	2.42 ± 0.12	3.02 ± 0.34	1.95 ± 0.07	1.40 ± 0.07	3.59 ± 0.70
**10a**	1.30 ± 0.09	2.98 ± 0.08	1.77 ± 0.14	1.25 ± 0.16	2.33 ± 0.28
**10b**	2.80 ± 0.36	3.33 ± 0.17	2.04 ± 0.13	0.82 ± 0.06	2.43 ± 0.13
**11a**	31.54 ± 18.66	96.35 ± 64.69	24.88 ± 3.17	31.76 ± 18.21	15.40 ± 6.22
**11b**	1.01 ± 0.06	0.35 ± 0.03	0.39 ± 0.03	0.89 ± 0.15	0.61 ± 0.04
**Amonafide**	4.81 ± 0.54	3.89 ± 0.28	2.60 ± 0.32	1.23 ± 0.07	5.10 ± 0.76

^a^ Cell growth inhibition was measured by the MTT assay, and values were expressed as mean IC_50_ of the triplicate experiment; ^b^ All these compounds (**6**, **7**, **10**, **11**) are in its di-hydrochloride forms.

We also screened all the synthetic intermediates in its mono-hydrochloride form against the same five cancer cell lines (Table 2). It was unexpected to find that compounds **8a** and **8b** which acted only as DNA-intercalators, exhibited close activities to **11b**. These results indicated that *N*-mustards can increase the cytotoxic activity of the naphathalimide derivatives, but not efficiently.

**Table 2:** IC_50_ values of synthetic intermediates over five cancer cell lines ^a^.

Compound ^b^	IC_5_ (μM)				
	HCT-116	PC-3	U87MG	Hep G2	SK-OV-3
**3a**	13.45 ± 0.88	45.82 ± 3.13	15.39 ± 1.39	22.60 ± 8.29	24.18 ± 4.64
**3b**	12.52 ± 1.47	49.17 ± 6.69	14.25 ± 1.19	12.17 ± 0.65	17.48 ± 1.68
**4a**	12.00 ± 0.46	25.54 ± 2.24	14.08 ± 0.86	9.43 ± 2.39	15.07 ± 2.17
**4b**	23.72 ± 2.53	67.16 ± 8.79	27.50 ± 5.02	18.80 ± 1.91	21.05 ± 3.09
**5a**	8.44 ± 0.68	7.40 ± 0.64	3.33 ± 0.37	4.20 ± 0.20	16.71 ± 1.47
**5b**	81.69 ± 3.79	64.16 ± 14.12	36.89 ± 13.15	31.19 ± 1.09	102.58 ± 6.99
**8a**	0.78 ± 0.06	1.83 ± 0.07	0.77 ± 0.02	0.71 ± 0.10	0.89 ± 0.03
**8b**	0.77 ± 0.05	1.38 ± 0.18	0.65 ± 0.02	0.56 ± 0.02	0.87 ± 0.03
**9b**	2.07 ± 0.24	14.60 ± 0.53	2.26 ± 0.37	1.04 ± 0.07	5.29 ± 0.31
**amonafide**	4.81 ± 0.54	3.89 ± 0.28	2.60 ± 0.32	1.23 ± 0.07	5.10 ± 0.76

^a^ Cell growth inhibition was measured by the MTT assay, and values were expressed as mean IC_50_ of the triplicate experiment. ^b^ All these compounds (**3**, **4**, **5**, **8**, **9**) are in its mono-hydrochloride forms.

**Figure 3:**
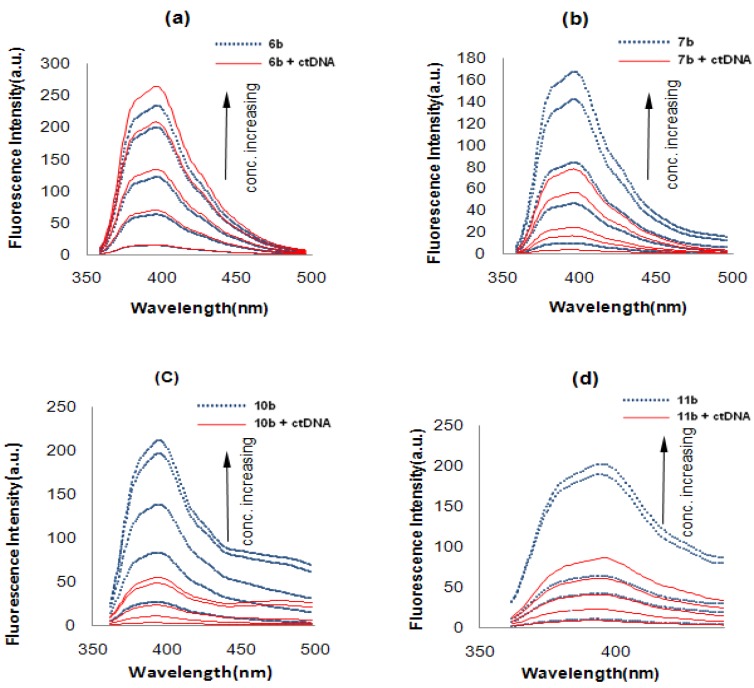
Fluorescence spectra of (**a**) **6b** (**b**) **7b** (**c**) **10b** (**d**) **11b**.

*Conditions:* Fluorescence spectra of (**a**) **6b** (**b**) **7b** (**c**) **10b** (**d**) **11b** (all in their dihydrochloride form) at different concentrations after incubated with ctDNA (50 μM). The concentrations of the four compounds were 1, 5, 10, 20 and 25 μM. The insets showed the relationship between the intensity of fluorescence and the concentration of the four compounds.

#### 2.2.2. Fluorescence Studies

A fluorescence study was employed to evaluate the interaction properties of the synthesized derivatives with DNA. Figure 3 illustrates the changes of fluorescence properties of compounds **6b**, **7b**, **10b** and **11b** before and after being mixed with calf thymus DNA (ctDNA).The solutions of various concentrations of compounds with ctDNA were incubated at 30 °C for 3 days, then the florescence spectra were recorded. We found that the fluorescence intensity of compound **6b** showed little change with or without ctDNA at all five concentrations, indicating a weak intercalating ability. On the contrary, compound **7b** exhibited an evident decrease of fluorescence intensity when ctDNA was added, which demonstrated the *N*-mustard is essential to increase the binding strength to DNA. Both compounds **10b** and **11b** showed obvious decreases of fluorescence intensity, but the difference between them is not significant. Those results indicate that: (1) an *N*-mustard residue is able to stabilize the DNA-compound complex after the intercalating process, and the binding potency is enhanced for *N*-mustard derivatives; (2) bis-naphthalimides **10b**, **11b** show generally much higher intercalating potential than mono-naphthalimides such as **6b**, **7b**, which is in accordance with previous research [19].

**Figure 4:**
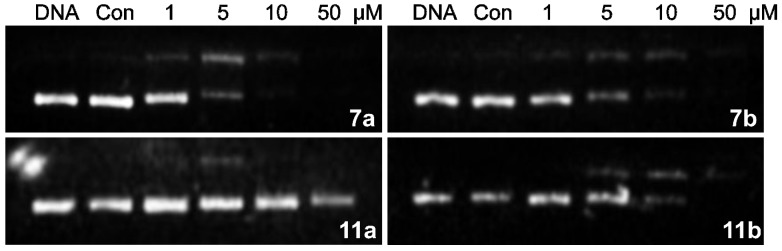
Agarose Gel Cross-Link Assay.

*Conditions:* compound and 2 μL supercoiled plasmid DNA (pUC-19, 0.16 μg/μL) were incubated at 37 °C for 1 h in Tris-HCl buffer (0.05 M, pH = 7.4). Con: precursor of each specific nitrogen mustard (**6a/b**, **10a/b**). Concentrations of drugs, from left to right: 1, 5, 10, 50 μM. All the compounds used in the assay are in their dihydrochloride form.

#### 2.2.3. DNA Interstrand Cross-Linking Assay

To evaluate the potency of DNA interstrand cross-linking brought by the *N*-mustard moiety [15], compounds **7a**, **b** and **11a**, **b** were selected to undergo an agarose gel cross-linking assay, at four concentration levels (Figure 4). Considering that naphthalimide chromophore can intercalate with DNA and so slow down DNA migration on the gel, **6a**,**b** and **10a**,**b** were employed, respectively, as controls for each mustine derivative. Compounds **7a**, **7b** and **11b** displayed DNA cross-linking potency at 1, 1 and 5 μM, respectively; while **11a** displayed almost no DNA cross-linking ability. The results for **7b**, **11a** and **11b** were consistent with their cytotoxic data. However, different from the cytotoxicity data, **7a** exhibited high DNA cross-linking activity, even at 1 μM concentration. The different performance of **7a** in cells and under agarose gel (with Tris-HCl buffer) conditions may result from its insufficient transmembrane access to the nucleus. The controls **10a** and **10b** displayed no DNA cross-linking abilities when compared with the *N*-mustards **11a** and **11b**, which showed similar activities in the previous tests. This result suggested the possibility that the *N*-mustard moiety did not show significant differences in *in vitro* tests, but played an efficient role in interacting with DNA.

#### 2.2.4. Flow Cytometry Assay

**Figure 5:**
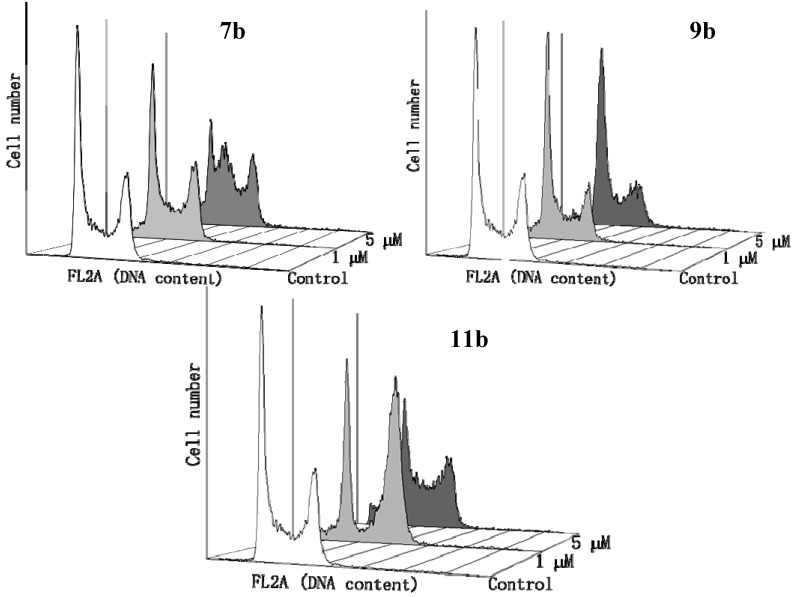
Effects of compounds on cell cycle distribution of HCT-116 cells.

Compounds that target DNA by intercalating or cross-linking often interfere with cellular DNA replication, thus inhibiting cell proliferation by inducing cell cycle arrest. To evaluate the effects of the compounds on cell cycle progression, the cell cycle distribution of HCT-116 cells was examined by flow cytometry after treatment with indicated concentrations of compounds **7b**, **9b** and **11b** for 24 h. As shown in Figure 5, compounds **7b** and **11b** induced G2/M and S phase cell cycle arrest, suggesting that these compounds could interfere with DNA synthesis. Compound **11b** showed a more pronounced arrest in G2/M phase at 1 μM than **7b**, which is in agreement with the higher cytotoxicity associated with **11b**. However, compound **9b**, a homo-*N*-mustard, exhibited marginal effects on the cell cycle distribution, even at 5 μM, implying the *N*-mustard moiety is essential for inhibition of DNA synthesis.

*Conditions:* Cells were incubated with indicated concentrations of **7b**, **9b**, **11b** for 24 h, then cells were stained by PI (20 μg/mL) and tested by flow cytometry.

#### 2.2.5. Immunoblotting Assay

**Figure 6:**
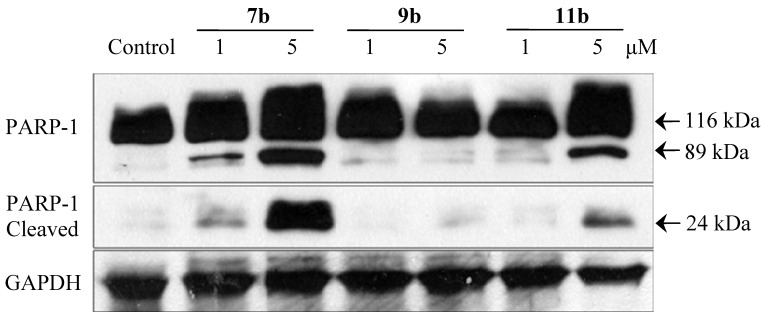
Effect of compounds on PARP-1 cleavage in HCT-116 cell.

Poly(ADP-ribose) polymerase (PARP-1) is specifically cleaved by caspases during the execution phase of apoptosis in response to DNA damage, thus its cleavage has been regarded as a biomarker of apoptosis [28]. Therefore, the cleavage of PARP-1 in HCT-116 cells treated with various compounds was detected by western blotting. As shown in Figure 6, both **7b** and **11b** induced significant cleavage of the original 116 kDa PARP-1 into 89 kDa and 24 kDa fragments, suggesting that these two compounds induced DNA-damage related apoptosis. In agreement with the results above, the homo-mustard **9b** showed no observable effect on the cleavage of PARP-1. Taken together, the above results suggest that compounds **7b** and **11b** induced DNA damage-related cell cycle arrest and apoptosis in HCT-116 cells, but **9b** might have a different mechanism of action.

*Conditions:* HCT-116 cells were treatment for 24 h in the presence of indicated concentrations of **7b**, **9b**, **11b**, and then cleavage of PARP1 was detected by immunoblotting. GAPDH, a house-keeping gene product, was used as loading control.

## 3. Experimental Section

### 3.1. General Information

All the solvents were of analytical grade. ^1^H-NMR and ^13^C-NMR spectra were obtained with JEOL-300 all examples below are 300 MHz but in Supplementary file spectra are labelled as 400 Mhz. Explain this confusing situation spectrometers. The chemical shifts were reported in ppm using TMS as internal standard. High resolution mass spectrometry was measured on a Bruker MicrOTOF-Q. Column chromatography was conducted on silica gel (200~300 mesh). Temperature range for rt is 25 ± 2 °C.

### 3.2. Synthesis

*2-(3-Hydroxypropyl)-1H-benzo[de]isoquinoline-1,3(2H)-dione* (**1**) [24]. A mixture of 1,8-naphthalic anhydride (4 g, 20.8 mmol) and 3-amino-1-propanol (1.86 mL, 24.6 mmol) in ethanol (40 mL) was heated under reflux for 5 h. The resulting mixture was concentrated by evaporating ethanol under reduced pressure to afford a solid residue, which was purified by column chromatography over silica gel (CH_2_Cl_2_/MeOH = 1:1) to afford compound **1** as an off-white solid (4.27 g, 83%).

*2-(3-Bromopropyl)-1H-benzo[de]isoquinoline-1,3(2H)-dione* (**2**) [25]. To a solution of compound **1** (2 g, 7.84 mmol) and NBS (2.12 g, 11.9 mmol) in dichloromethane (20 mL, dry) was added PPh_3_ (2.49 g, 8.2 mmol) in portions in an ice bath. The resulting solution was kept at room temperature overnight, and then purified by column chromatography over silica gel (petroleum ether/CH_2_Cl_2_ = 3:1) to afford compound **2** (2.25 g, 90%).

*N-[2-(1,3-Dioxo-2,3-dihydro-1H-benz[de]-isoquinolin-2-yl)propyl]-ethanolamine* (**3a**). A mixture of compound **2** (1 g, 3.15 mmol), ethanolamine (2 mL, 33.1 mmol) and K_2_CO_3_ (1.8 g, 13.0 mmol) in 10 mL of CH_3_CN was heated at 70 °C for 30 h. The resulting mixture was filtered to remove the K_2_CO_3_ residue, condensed *in vacuo*, then dissolved in H_2_O (80 mL) and extracted with CH_2_Cl_2_ (100 mL). The extract was dried over Na_2_SO_4_ and purified by column chromatography (CH_2_Cl_2_/MeOH = 20:1~10:1) to yield pure **3a** (0.79 g, 80%) as a faint yellow solid. ^1^H-NMR (300 MHz, CDCl_3_) δ_H_ (ppm): 1.94–2.01 (m, 2H), 2.69–2.76 (m, 4H, NHCH_2_ and OH), 2.82 (t, 2H, *J* = 5.1 Hz), 3.66 (t, 2H, *J* = 5.1 Hz), 4.29 (t, 2H, *J* = 6.9 Hz), 7.75 (dd, 2H, *J* = 7.5, 8.1 Hz), 8.21 (d, 2H, *J* = 8.4 Hz), 8.59 (d, 2H, *J* = 7.2 Hz). ^13^C-NMR (75 MHz, CDCl_3_): δ 164.3(2C), 134.0 (2C), 131.3 (4C), 126.9 (2C), 122.5 (2C), 60.6, 50.9, 46.2, 38.1, 28.3.HRMS (ESI): Calcd for C_17_H_19_N_2_O_3_: 299.1390 [M+H]^+^, Found: 299.1383 [M+H]^+^.

*N-[2-(1,3-Dioxo-2,3-dihydro-1H-benz[de]-isoquinolin-2-yl)propyl]-3-amino-1-propanol* (**3b**). Compound **3b** was synthesized by a similar procedure as the synthesis of **3a**. Compound **2** (1 g, 3.15 mmol) and 3-amino-1-propanol (2 mL, 27.1 mmol) were employed to produce **3b** (yellow oil, 0.89 g, 91%). ^1^H-NMR (300 MHz, CDCl_3_) δ_H_ (ppm): 1.73–1.76 (m, 2H), 1.95–2.00 (m, 2H), 2.73 (t, 2H, *J* = 6.6 Hz), 2.90 (t, 1H, *J* = 5.4 Hz, NH), 3.46–3.47 (m, 3H, CH_2_N, OH), 3.83 (t, 2H, *J* = 5.1 Hz), 4.24 (t, 2H, *J* = 6.3 Hz), 7.71–7.76 (m, 2H), 8.19 (d, 2H, *J* = 7.2 Hz), 8.70 (d, 2H, *J* = 6.0 Hz). ^13^C-NMR (75 MHz, CDCl_3_): δ 164.2 (2C), 133.9 (2C), 131.4, 131.2 (3C), 126.8 (2C), 122.3 (2C), 63.7, 49.2, 46.7, 37.9, 30.6, 27.9; HRMS (ESI): Calcd for C_18_H_21_N_2_O_3_: 313.1547 [M+H]^+^, Found: 313.1555 [M+H]^+^.

*N-[2-(1,3-Dioxo-2,3-dihydro-1H-benz[de]-isoquinolin-2-yl)propyl]-N-methylethanolamine* (**4a**). To formaldehyde (37%, 1.2 mL) was added formic acid (5 mL) dropwise in an ice bath, and the resulting solution was added into compound **3a** (711 mg, 2.39 mmol) and stirred for 10 min in an ice bath, then heated to 105 °C overnight. Removal of residual formaldehyde and formic acid under reduced pressure gave crude product, which was dissolved in water and treated with Na_2_CO_3_ to pH > 7, and extracted with CH_2_Cl_2_. The organic layer was dried with Na_2_SO_4_ and purified by column chromatography (CHCl_3_/MeOH = 30:1) to yield compound **4a** (yellow oil, 0.64 g, 85.6%). ^1^H-NMR (300 MHz, CDCl_3_) δ_H_ (ppm): 1.90–1.99 (m, 2H), 2.29 (s, 3H), 2.54–2.59 (m, 4H), 3.02 (s, 1H), 3.61 (t, 2H, *J* = 5.1 Hz), 7.75 (dd, 2H, *J* = 7.5, 8.1 Hz), 8.21 (d, 2H, *J* = 8.4 Hz), 8.59–8.61 (m, 2H). ^13^C-NMR (75 MHz, CDCl_3_): δ 164.2 (2C), 133.9 (2C), 131.5, 131.2 (3C), 126.9 (2C), 122.6 (2C), 59.0, 58.5, 55.2, 41.4, 38.5, 25.7; HRMS (ESI): Calcd for C_18_H_21_N_2_O_3_: 313.1547 [M+H]^+^, Found: 313.1560 [M+H]^+^.

*N-[2-(1,3-Dioxo-2,3-dihydro-1H-benz[de]-isoquinolin-2-yl)propyl]-N-methyl-3-amino-1-propanol* (**4b**). Compound **4b** was synthesized by a similar procedure as the synthesis of **4a**. Compound **3b** (852 mg, 2.72 mmol) was employed to produce **4b** (yellow oil, 0.68 g, 77%). ^1^H-NMR (300 MHz, CDCl_3_) δ_H_ (ppm): 1.70–1.77 (m, 2H), 1.90–2.00 (m, 2H), 2.31 (s, 3H), 2.55 (t, 2H, *J* = 7.5 Hz), 2.64 (t, 2H, *J* = 5.7 Hz), 3.84 (t, 2H, *J* = 5.4 Hz), 4.22 (t, 2H, *J* = 7.5 Hz), 5.30 (s, 1H, OH), 7.75 (dd, 2H, *J* = 8.4, 7.2 Hz), 8.21 (d, 2H, *J* = 8.4 Hz), 8.59 (d, 2H, *J* = 7.2 Hz). ^13^C-NMR (75 MHz, CDCl_3_): δ 164.1 (2C), 133.9 (2C), 131.5, 131.2 (3C), 126.9 (2C), 122.5 (2C), 64.3, 57.8, 55.9, 41.8, 38.5, 27.8, 25.6; HRMS (ESI): Calcd for C_19_H_23_N_2_O_3_: 327.1703 [M+H]^+^, Found: 327.1704 [M+H]^+^.

*N-[2-(1,3-Dioxo-2,3-dihydro-1H-benz[de]-isoquinolin-2-yl)propyl]-N-methyl-2-chloroethanamine* (**5a**). To a solution of compound **4a** (579 mg, 1.86 mmol) in CHCl_3_ (30 mL) was added SOCl_2_ (1 mL) dropwise and the mixture was stirred at room temperature for 48 h. Removal of the solvent under reduced pressure gave the crude product, which was dissolved in water, treated with Na_2_CO_3_ until pH > 7, and extracted with CH_2_Cl_2_. The organic layer was dried with Na_2_SO_4_ and purified by column chromatography (CHCl_3_/MeOH = 50:1) to yield compound **5a** (pale yellow solid, 80.0%). ^1^H-NMR (300 MHz, CDCl_3_) δ_H_ (ppm): 1.87–1.97 (m, 2H), 2.32 (s, 3H), 2.59 (t, 2H, *J* = 7.2 Hz), 2.74 (t, 2H, *J* = 7.2 Hz), 3.56 (t, 2H, *J* = 7.2 Hz), 4.23 (t, 2H, *J* = 7.5 Hz), 7.75 (dd, 2H, *J* = 7.8, 7.5 Hz), 8.21 (d, 2H, *J* = 8.1 Hz), 8.59 (d, 2H, *J* = 7.2 Hz). ^13^C-NMR (75 MHz, CDCl_3_): δ 164.2 (2C), 133.9 (2C), 131.6, 131.2, 131.1 (2C), 126.9 (2C), 122.7 (2C), 58.9, 55.3, 42.1, 41.6, 38.7, 25.7; HRMS (ESI): Calcd for C_18_H_20_ClN_2_O_2_: 331.1208 [M+H]^+^, Found: 331.1219 [M+H]^+^.

*N-[2-(1,3-Dioxo-2,3-dihydro-1H-benz[de]-isoquinolin-2-yl)propyl]-N-methyl-3-chloro-1-propylamine* (**5b**). Compound **5b** was synthesized by a similar procedure as the synthesis of **5a**. Compound **4b** (625 mg, 1.92 mmol) was employed to produce **5b** (light yellow solid, 0.55 g, 82.9%). ^1^H-NMR (300 MHz, CDCl_3_) δ_H_ (ppm): 2.36–2.39 (brs, 4H), 3.78 (s, 3H), 3.16 (brs, 4H), 3.67 (t, 2H, *J* = 5.4 Hz), 4.32 (t, 2H, *J* = 6.9 Hz), 7.78 (t, 2H, *J* = 7.8 Hz), 8.25 (d, 2H, *J* = 8.4 Hz), 8.60 (d, 2H, *J* = 7.2 Hz). ^13^C-NMR (75 MHz, CDCl_3_): δ 164.2 (2C), 134.4 (2C), 131.6 (4C), 128.1, 127.0, 122.1 (2C), 54.3, 54.1, 41.8, 40.1, 37.6, 26.9, 22.9; HRMS (ESI): Calcd for C_19_H_22_ClN_2_O_2_: 345.1364 [M+H]^+^, Found: 345.1361 [M+H]^+^.

*N-[2-(1,3-Dioxo-2,3-dihydro-1H-benz[de]-isoquinolin-2-yl)propyl]-N-methyl-N',N'-bis(2-hydroxyethyl)-ethylenediamine* (**6a**). A mixture of compound **5a** (431 mg, 1.31 mmol), K_2_CO_3_ (300 mg, 2.17 mmol), KI (100 mg, 0.60 mmol) and diethanolamine (0.8 mL) in CH_3_CN (15 mL) was stirred at 80 °C under N_2_ for 72 h. The resulting mixture was evaporated under reduced pressure to give the crude product. The residue was dissolved in water (50 mL) and extracted with CH_2_Cl_2_. The organic layer was dried with Na_2_SO_4_ and purified by column chromatography (CHCl_3_/MeOH = 30:1~10:1) to yield compound **6a ** (yellow oil, 58%). ^1^H-NMR (300 MHz, CDCl_3_) δ_H_ (ppm): 1.93–1.98 (m, 2H), 2.28 (s, 3H), 2.49–2.71 (m, 10H), 3.58 (m, 4H), 4.20 (t, 2H, *J* = 7.5 Hz), 4.34 (brs, 2H, OH), 7.70–7.75 (m, 2H), 8.18 (d, 2H, *J* = 8.1 Hz), 8.56 (d, 2H, *J* = 7.2 Hz). ^13^C-NMR (75 MHz, CDCl_3_): δ 164.0 (2C), 133.8 (2C), 131.4, 131.1 (3C), 126.8 (2C), 122.4 (2C), 59.9 (2C), 57.3 (2C), 56.1, 55.1, 51.7, 41.5, 38.6, 25.0; HRMS (ESI): Calcd for C_22_H_30_N_3_O_4_: 400.2231 [M+H]^+^, Found: 400.2241 [M+H]^+^.

*N-[2-(1,3-Dioxo-2,3-dihydro-1H-benz[de]-isoquinolin-2-yl)propyl]-N-methyl-N',N'-bis(2-hydroxyethyl)-1,3-diamine* (**6b**). Compound **6b** was synthesized by a similar procedure as the synthesis of **6a**. Compound **5b** (451 mg, 1.31 mmol) was employed to produce **6b** (yellow oil, 91.2%). ^1^H-NMR (300 MHz, CDCl_3_) δ_H_ (ppm): 1.66 (t, 2H, *J* = 6.3 Hz), 1.95 (t, 2H, *J* = 6.9 Hz), 2.25 (s, 3H), 2.47–2.65 (m, 10H), 3.63 (t, 3H, *J* = 5.1 Hz), 3.99 (brs, 2H), 4.21 (t, 2H, *J* = 7.5 Hz), 7.74 (t, 2H, *J* = 7.5 Hz), 8.20 (d, 2H, *J* = 8.1 Hz), 8.58 (d, 2H, *J* = 7.5 Hz). ^13^C-NMR (75 MHz, CDCl_3_): δ 164.1 (2C), 133.9 (2C), 131.4, 131.1 (2C), 128.0, 126.9 (2C), 122.5 (2C), 59.9 (2C), 56.1 (2C), 54.9, 54.6, 52.0, 41.9, 38.8, 25.0, 24.4; HRMS (ESI): Calcd for C_23_H_32_N_3_O_4_: 414.2387 [M+H]^+^, Found: 414.2384 [M+H]^+^.

*N-[2-(1,3-Dioxo-2,3-dihydro-1H-benz[de]-isoquinolin-2-yl)propyl]-N-methyl-N',N'-bis(2-chloroethyl)-ethylenediamine* (**7a**). The solution of compound **6a** (75 mg, 0.19 mmol) in SOCl_2_ (2 mL) was stirred at room temperature overnight. The remaining SOCl_2_was completely removed by adding Et_2_O and then condensing the mixture several times *in vacuo*. The residue was dissolved in CH_2_Cl_2_; by adding Et_2_O into the solution, a white solid was formed and a gray solid was obtained by filtration with a yield of 85%. The purity of **7a** as determined by NMR meets the requirements for the cytotoxicity assay. ^1^H- NMR (300 MHz, D_2_O) δ_H_ (ppm): 1.93 (brs, 2H), 2.88 (s, 3H), 3.46–3.86 (m, 14H), 7.24–7.30 (m, 2H), 7.72–7.78 (m, 4H). ^13^C-NMR (75 MHz, D_2_O): δ165.2 (2C), 135.8 (2C), 131.9, 130.9 (3C), 127.5 (2C), 120.3 (2C), 55.6(4C), 51.5, 48.4, 40.7, 39.1, 37.6, 24.9.HRMS (ESI): Calcd for C_22_H_28_Cl_2_N_3_O_2_: 436.1553 [M+H]^+^, Found: 436.1559 [M+H]^+^.

*N-[2-(1,3-Dioxo-2,3-dihydro-1H-benz[de]-isoquinolin-2-yl)propyl]-N-methyl-N',N'-bis(2-chloroethyl)-1,3-diamine* (**7b**). To a solution of compound **6b** (123 mg, 0.30 mmol) in CHCl_3_ (20 mL) was added SOCl_2_ (0.15 mL) dropwise, and the solution became turbid. After the reaction was kept at 65 °C for 4 h, the solution became clear. The solvent was removed *in vacuo* to give the crude product, which was dissolved in conc. NaHCO_3_ solution and extracted with CH_2_Cl_2_. The organic layer was washed by conc. NaCl, dried by Na_2_SO_4_ and purified by column chromatography (CHCl_3_/MeOH = 50:1) to yield 7b (yellow oil, 45%) ^1^H-NMR (300 MHz, CDCl_3_) δ_H_(ppm): 1.62 (t, 2H, *J* = 7.2 Hz), 1.92–1.99 (m, 2H), 2.27 (s, 3H), 2.43 (t, 2H, *J* = 6.6 Hz), 2.52–2.62 (m, 4H), 2.84 (t, 4H, *J* = 7.2 Hz), 3.50 (t, 4H, *J* = 6.9 Hz), 4.22 (t, 2H, *J* = 7.5 Hz), 7.74 (t, 2H, *J* = 7.8 Hz), 8.19 (d, 2H, *J* = 7.8 Hz), 8.57 (d, 2H, *J* = 7.2 Hz). ^13^C-NMR (75 MHz, CDCl_3_): δ 164.0 (2C), 133.8 (2C), 131.4, 131.1 (3C), 127.9, 126.8, 122.5 (2C), 56.3 (2C), 55.2, 54.9, 52.4, 41.9 (2C), 41.7, 38.7, 25.4, 25.2; HRMS (ESI): Calcd for C_23_H_30_Cl_2_N_3_O_2_: 450.1710 [M+H]^+^, Found:450.1716 [M+H]^+^.

*N,N-Bis[2-(1,3-dioxo-2,3-dihydro-1H-benz[de]-isoquinolin-2-yl)propyl]ethanolamine* (**8a**). A mixture of compound **2** (318 mg, 1 mmol), ethanolamine (30 μL, 0.5 mmol) and K_2_CO_3_ (552 mg, 4 mmol) in CH_3_CN (10 mL) was heated at 80 °C in and oil bath under N_2_ for 24 h. The resulting mixture was filtrated to remove the solid salt and concentrated *in vacuo*, then purified by column chromatography over silica gel (CH_2_Cl_2_/MeOH = 30:1~20:1) to yield **8a** (light yellow solid, 174 mg, 65%). ^1^H-NMR (300 MHz, CDCl_3_) δ_H_(ppm): 1.87–1.96 (m, 4H), 2.64–2.69 (m, 6H), 3.63 (m, 2H), 4.24 (t, 4H, *J* = 7.5 Hz), 7.71 (t, 4H, *J* = 7.5 Hz), 8.16 (d, 4H, *J* = 8.1 Hz), 8.54 (d, 4H, *J* = 7.2 Hz). ^13^C-NMR (75 MHz, CDCl_3_): δ 164.0 (4C), 133.8 (4C), 131.4 (2C), 131.1 (4C), 128.0 (2C), 126.8 (4C), 122.6 (4C), 59.1, 56.3, 51.6 (2C), 38.6 (2C), 25.7 (2C); HRMS (ESI): Calcd for C_32_H_30_N_3_O_5_: 536.2180 [M+H]^+^, Found:536.2184 [M+H]^+^.

*N,N-Bis[2-(1,3-dioxo-2,3-dihydro-1H-benz[de]-isoquinolin-2-yl)propyl]-3-amino-1-propanol* (**8b**). Compound **8b** was synthesized by a similar procedure as the synthesis of **8a**. Compound **2** (318 mg, 1 mmol), 3-amino-1-propanol (0.037 mL, 0.5 mmol) and K_2_CO_3_ (552 mg, 4 mmol) were employed to produce **8b** (light yellow oil, 137 mg, 50%). ^1^H-NMR (300 MHz, CDCl_3_) δ_H_(ppm): 1.76 (m, 2H), 1.95 (m, 4H), 2.64–2.72 (m, 6H), 3.88 (t, 2H), 4.21 (t, 4H, *J* = 7.5 Hz), 7.69 (t, 4H, *J* = 7.2 Hz), 8.15 (d, 4H, *J* = 8.4 Hz), 8.49 (d, 4H, *J* = 7.5 Hz). ^13^C-NMR (75 MHz, CDCl_3_):δ 163.9 (4C), 133.7 (4C), 131.3 (2C), 130.9 (4C), 127.8 (2C), 126.7 (4C), 122.3 (4C), 62.9, 52.7, 51.6 (2C), 38.7 (2C), 28.4, 25.2 (2C); HRMS (ESI): Calcd for C_33_H_32_N_3_O_5_: 550.2336 [M+H]^+^, Found: 550.2346 [M+H]^+^.

*N,N-Bis[2-(1,3-dioxo-2,3-dihydro-1H-benz[de]-isoquinolin-2-yl)propyl]-3-chloro-1-propylamine* (**9b**). To a solution of compound **8b** (1.100 g, 2.0 mmol) in CHCl_3_ (20 mL) was added SOCl_2_ (0.8 mL) dropwise and the mixture was stirred at room temperature for 2 days. The resulting mixture was concentrated under reduced pressure to give a yellow solid residue. The residue was rapidly purified by column chromatography over silica gel (CH_2_Cl_2_/MeOH = 40:1) to yield **9b** (light yellow solid, 87.5%). Compound **9b** was easily transferred into the corresponding ammonium salt, so it should be used immediately for next step. ^1^H-NMR (300 MHz, CDCl_3_) δ_H_ (ppm): 1.88–2.04 (m, 6H), 2.64 (t, 6H, *J* = 6.3 Hz), 3.69 (t, 2H, *J* = 6.3 Hz), 4.21 (t, 4H, *J* = 6.6 Hz), 7.68 (t, 4H, *J* = 7.2 Hz), 8.26 (d, 4H, *J* = 8.1 Hz), 8.77 (d, 4H, *J* = 7.2 Hz). ^13^C-NMR (75 MHz CDCl_3_): δ 163.8 (4C), 133.6 (4C), 131.3 (2C), 130.9 (4C), 127.8 (2C), 126.7 (4C), 122.5 (4C), 51.6, 50.7 (2C), 43.4, 38.8 (2C), 30.5, 25.5 (2C); HRMS (ESI): Calcd for C_33_H_30_ClN_3_O_4_: 568.2003 [M+H]^+^, Found: 568.1982 [M+H]^+^.

*N,N-Bis[2-(1,3-dioxo-2,3-dihydro-1H-benz[de]-isoquinolin-2-yl)propyl]-N',N'-bis(2-hydroxyethyl)-ethylenediamine* (**10a**). The synthesis of compound **10a** was divided into two parts. Firstly, compound **8a** (560 mg, 1.05 mmol) was employed to produce **9a** through a similar procedure as **9b**. Then compound **9a** was directly used for the next step. A mixture of **9a** (350 mg, 0.63 mmol), K_2_CO_3_ (540 mg, 3.91 mmol), KI (100 mg, 0.60 mmol) and diethanolamine (0.8 mL) in CH_3_CN (10 mL) was refluxed under N_2_ for 60 h. The resulting mixture was filtrated to remove solid K_2_CO_3_, and then concentrated under reduced pressure. The crude product was purified by column chromatography over silica gel (CHCl_3_/MeOH = 30:1) to yield 10a (light yellow oil, 86.0%).^1^H-NMR (300 MHz, CDCl_3_) δ_H _ (ppm): 1.94 (t, 4H, *J* = 6.9 Hz), 2.62–2.70 (m, 12H), 3.61 (brs, 6H), 4.22 (t, 4H, *J* = 6.6 Hz), 7.72 (t, 4H, *J* = 7.2 Hz), 8.18 (d, 4H, *J* = 8.1 Hz), 8.54 (d, 4H, *J* = 7.2 Hz). ^13^C-NMR (75 MHz, CDCl_3_): δ 164.1 (4C), 133.8 (4C), 131.4 (2C), 131.1 (6C), 126.8 (4C), 122.6 (4C), 59.9 (2C), 57.2 (2C), 52.6, 52.4, 51.4 (2C), 38.9 (2C), 24.8 (2C); HRMS (ESI): Calcd for C_36_H_39_N_4_O_6_: 536.2180 [M+H]^+^, Found:536.2848 [M+H]^+^.

*N,N-Bis[2-(1,3-dioxo-2,3-dihydro-1H-benz[de]-isoquinolin-2-yl)propyl]-N',N'-bis(2-hydroxyethyl)-1,3-diamine* (**10b**). Compound **10b** was synthesized by a similar procedure as the synthesis of **10a**. Compound **8b** (1.000 g, 1.8 mmol) was employed to produce **9b**, which was then used immediately for the next step to yield **10b** (light yellow oil, 81.6%). ^1^H-NMR (300 MHz, CDCl_3_) δ_H_ (ppm): 1.68–1.73 (t, 2H), 1.89–1.96 (m, 4H), 2.56–2.68 (m, 12H), 3.38 (brs, 2H), 3.64–3.67 (t, 4H), 4.22 (t, 4H, *J* = 7.5 Hz), 7.72 (t, 4H, *J* = 7.5 Hz), 8.17 (d, 4H, *J* = 7.8 Hz), 8.55 (d, 4H, *J* = 7.5 Hz). ^13^C-NMR (75 MHz, CDCl_3_): δ164.2 (4C), 133.8 (4C), 131.5 (2C), 131.2 (4C), 128.0 (2C), 126.9 (4C), 122.6 (4C), 59.8 (2C), 56.6 (2C), 52.8, 51.8 (2C), 51.1, 39.0 (2C), 25.3 (2C), 24.8; HRMS (ESI): Calcd for C_37_H_41_N_4_O_6_: 637.3021 [M+H]^+^, Found: 637.3027 [M+H]^+^.

*N,N-Bis[2-(1,3-dioxo-2,3-dihydro-1H-benz[de]-isoquinolin-2-yl)propyl]-N',N'-bis(2-chloroethyl)–ethylenediamine hydrochloride* (**11a**). The solution of compound **10a** (50 mg, 0.08 mmol) in SOCl_2_ (1 mL) was stirred at room temperature overnight. The remaining SOCl_2_ was completely removed by adding Et_2_O and then condensed for several times *in vacuo*. The residue was dissolved in a mixed solution of methanol and a little methylene chloride, then a drop of concentrated hydrochloric acid was added to afford a white solid precipitate. The suspension was filtered to yield crude product **11a** hydrochloride as white solid (30 mg, 51.0%). ^1^H-NMR (300 MHz, DMSO-*d*_6_) δ_H_(ppm): 2.09 (brs, 4H), 3.07–3.77 (brs, 16H), 4.09 (t, 4H, *J* = 6.3 Hz), 7.82 (t, 4H, *J* = 7.8 Hz), 8.36–8.44 (m, 8H). ^13^C- NMR (75 MHz, DMSO-*d*_6_): δ 163.7 (4C), 134.5 (4C), 131.3 (2C), 130.8 (4C), 127.4 (2C), 127.2 (4C), 122.1 (4C), 54.4 (2C), 50.3 (2C), 47.6 (2C), 37.2 (4C), 22.1 (2C); HRMS (ESI): Calcd for C_36_H_37_Cl_2_N_4_O_4_: 659.2186 [M+H]^+^, Found:659.2180 [M+H]^+^.

*N,N-Bis[2-(1,3-dioxo-2,3-dihydro-1H-benz[de]-isoquinolin-2-yl)propyl]-N',N'-bis(2-chloroethyl)-1,3-diamine* (**11b**). To a solution of compound **10b** (190 mg, 0.30 mmol) in CHCl_3_ (25 mL) was added SOCl_2_ (0.15 mL) dropwise and the mixture stirred at 65 °C for 2 h. The solvent was removed under reduced pressure, then water (20 mL) and solid Na_2_CO_3_ were added to neutralize the solution, which was extracted with CH_2_Cl_2_ (50 mL). The organic layer was washed with saturated NaCl, dried over MgSO_4_, concentrated and then purified by column chromatography over silica gel (CHCl_3_/MeOH = 50:1) to yield **11b** (yellow oil, 77.0%). ^1^H-NMR (300 MHz, CDCl_3_) δ_H_ (ppm): 1.65 (brs, 2H), 1.92 (brs, 4H), 2.63–2.67 (m, 8H), 2.86 (t, 4H, *J* = 7.2 Hz), 3.51 (t, 4H, *J* = 7.2 Hz), 4.23 (t, 4H, *J* = 7.2 Hz), 7.72 (t, 4H, *J* = 7.8 Hz), 8.18 (d, 4H, *J* = 7.8 Hz), 8.54 (d, 4H, *J* = 7.2 Hz). ^13^C-NMR (75 MHz, CDCl_3_): δ 164.0 (4C), 133.7 (4C), 131.5 (2C), 131.1 (4C), 128.0 (2C), 126.8 (4C), 122.6 (4C), 56.4 (2C), 52.7, 51.6 (2C), 51.3, 42.0 (2C), 38.8 (2C), 25.2 (3C); HRMS (ESI): Calcd for C_37_H_39_Cl_2_N_4_O_4_: 673.2343 [M+H]^+^, Found: 673.2341 [M+H]^+^.

### 3.3. Fluorescence Study Experiment

Each compound was divided into two groups: one was added with a constant concentration of ctDNA (calf thymus DNA, 50 μM) and the other was without the ctDNA as control. Various concentrations of compounds ranging from 1 μM to 25 μM (1, 5, 10, 20 and 25 μM) were used. To every sample was added Tris-HCl buffer (25 mM, pH = 7.4) to get a final volume of 4 mL, which was stirred constantly at 30 °C in the dark for 3 days before recording each spectrum.

### 3.4. Agarosegel Cross-Linking Assay

Each compound was tested at four concentrations (1, 5, 10, 50 μM). To each sample was added 2 μL plasmid DNA (PUC-19, 0.16 μg/μL) and various concentrations of compounds, diluted by Tris-HCl buffer (0.05 M, pH = 7.4) to reach a final volume of 15 μL. Every group has a pure DNA sample as blank control and its own control sample by using its own precursor in concentration of 10 μM. All the above samples were incubated at 37 °C for 1 h and separated on an agarose gel (120 V, 45 min).

### 3.5. In Vitro Cytotoxicity Evaluation

All the compounds were tested in their mono- or dihydrochloride form. The samples were prepared by adding a stoichiometric amount of 1 M HCl to the solution, and then removing the solvent to obtain the target compounds. All cells used in the research were prepared at 3.5 × 10^4^ cells/mL concentration and each 100 μL cells suspension was seeded in 96-well cell incubated for 24 h (37 °C, 5% CO_2_). Then each solution was added and incubated for another 72 h. For the control group, equivalent concentration of DMSO (final concentration 0.5%) was added. MTT (3-[4,5-dimethylthiazol-2yl]-diphenyltetrazolium bromide) method was employed to measure the number of surviving cells and the OD value was recorded at 492 nm/620 nm. The IC_50_ values were calculated using Prism Graphpad software of the triplicate experiment.

### 3.6. Flow Cytometry Assay

Cells were trypsinized and washed twice with PBS, then fixed in 75% alcohol for at least 30 min at 4 °C. The fixed cells were recovered by centrifugation and washed twice with PBS, and then stained with PI in PBS containing RNAase and 0.1% Triton-X100 for 30 min at 37 °C. The cellular DNA content was measured by a FACScalibur flow cytometer (BD Bioscience, San Jose, CA, USA).

### 3.7. Immunoblotting Assay

Cells were lysed using RIPA lysis buffer containing PMSF and phosphatase/protease inhibitors. The total cellular protein extracts was qualified by BCA assay. 20 μg of total protein was resolved on 10% SDS-PAGE, then electro-transferred onto PVDF membranes, and incubated with appropriate antibodies overnight at 4 °C, then washed in PBS containing 0.1% Tween 20 and incubated with corresponding secondary antibodies for 1 h at room temperature. After that the membranes were developed with the enhanced chemiluminescence Western Blotting detection reagent (Amersham-Pharmacia, Piscataway, NJ, USA).

## 4. Conclusions

A series of mono- and bisnaphthalimide *N*-mustard derivatives were synthesized as potent antitumor agents. Through conjugating *N*-mustard to naphthalimide, improved cytotoxicity was obtained. Compound **11b** exhibited considerably cytotoxicity compared with amonafide, and showed potency as an antitumor agent. Even though more investigation is still needed to evaluate the efficiency of the *N*-mustard moiety, the bioactivity results suggest that the length of the flexible amine link and naphthalimide core could improve the cytotoxicity. Our work is meaningful in compensating for the lack of research in this field of combining *N*-mustards with naphthalimides.

## Supplementary Materials

Supplementary materials can be accessed at:  http://www.mdpi.com/1420-3049/19/7/8803/s1.
