# Supplementary material for: Synthesis and Cytotoxicity Evaluation of Naphthalimide Derived N-Mustards
Source: Molecules. 2014 Jun 25;19(7):8803–19. doi: 10.3390/molecules19078803 (PMC6271267; doi:10.3390/molecules19078803)

Support Information

Table of Contents

^{1}H NMR and ^{13}C NMR spectra of compound **3a**……………………………….……S2
^{1}H NMR and ^{13}C NMR spectra of compound **4b**……………………………….……S3
^{1}H NMR and ^{13}C NMR spectra of compound **5a**……………………………….……S4
^{1}H NMR and ^{13}C NMR spectra of compound **5b**……………………………….……S5
^{1}H NMR and ^{13}C NMR spectra of compound **6a**……………………………….……S6
^{1}H NMR and ^{13}C NMR spectra of compound **6b** ……………….………..…..……S7
^{1}H NMR and ^{13}C NMR spectra of compound **7a**……………………………….……S8
^{1}H NMR and ^{13}C NMR spectra of compound **7b**……………………………….……S9
^{1}H NMR and ^{13}C NMR spectra of compound **8a**……………………………….……S10
^{1}H NMR and ^{13}C NMR spectra of compound **8b**……………………………….……S11
^{1}H NMR and ^{13}C NMR spectra of compound **9a**……………………………….……S12
^{1}H NMR and ^{13}C NMR spectra of compound **9b**……………………………….……S13
^{1}H NMR and ^{13}C NMR spectra of compound **10a**…………………………….…..S14
^{1}H NMR and ^{13}C NMR spectra of compound **10b**…………………………….…..S15
^{1}H NMR and ^{13}C NMR spectra of compound **11a**…………………………….…..S16
^{1}H NMR and ^{13}C NMR spectra of compound **11b**…………………………….…..S17

3a ^{1}H-NMR (300 MHz, $CDCl_3$).

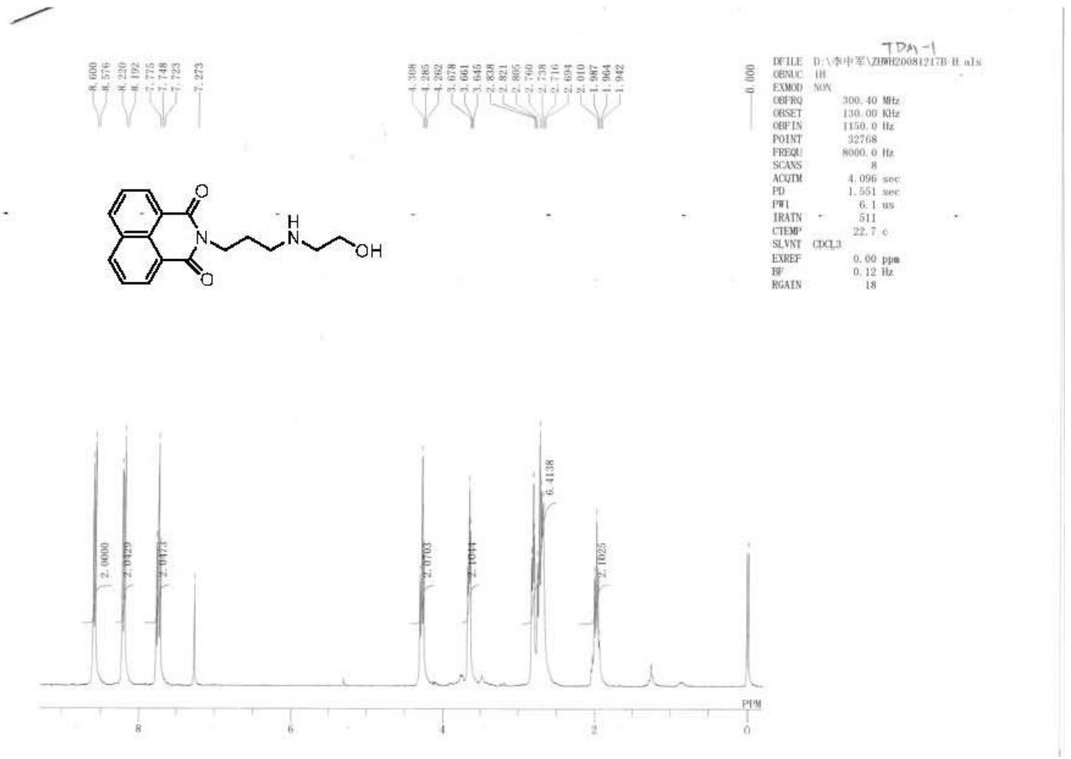

3a ^{13}C-NMR (75 MHz, $CDCl_3$).

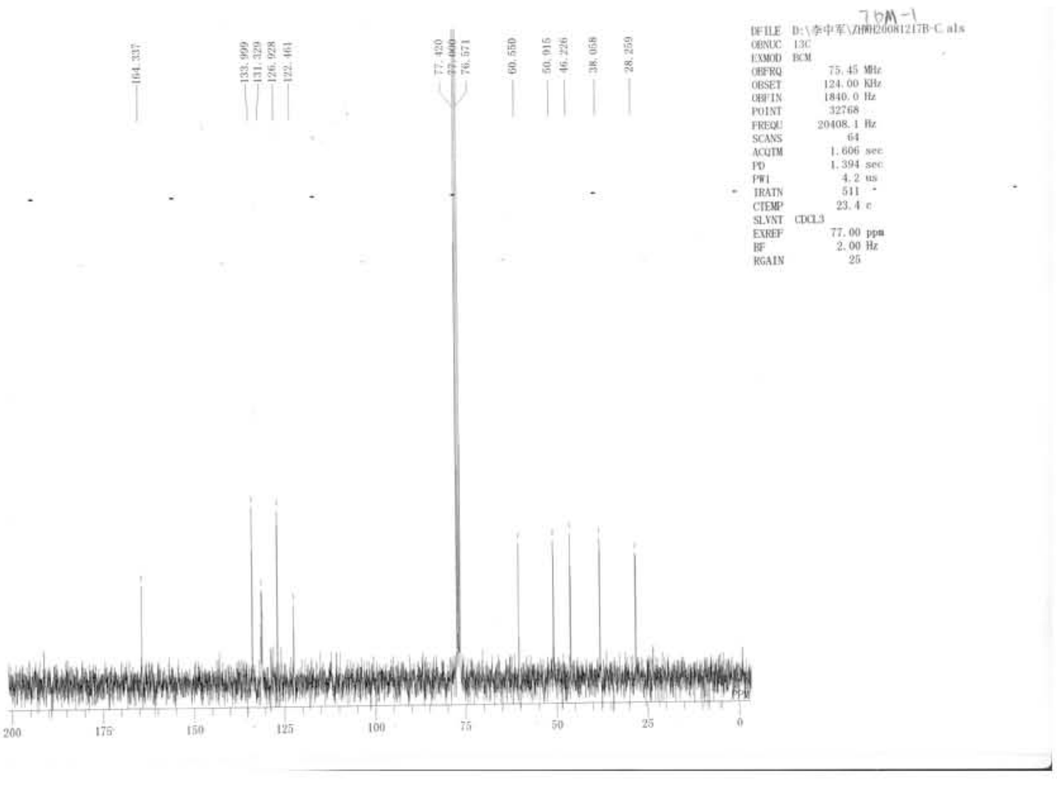

3b ^{1}H-NMR (300 MHz, $CDCl_3$).

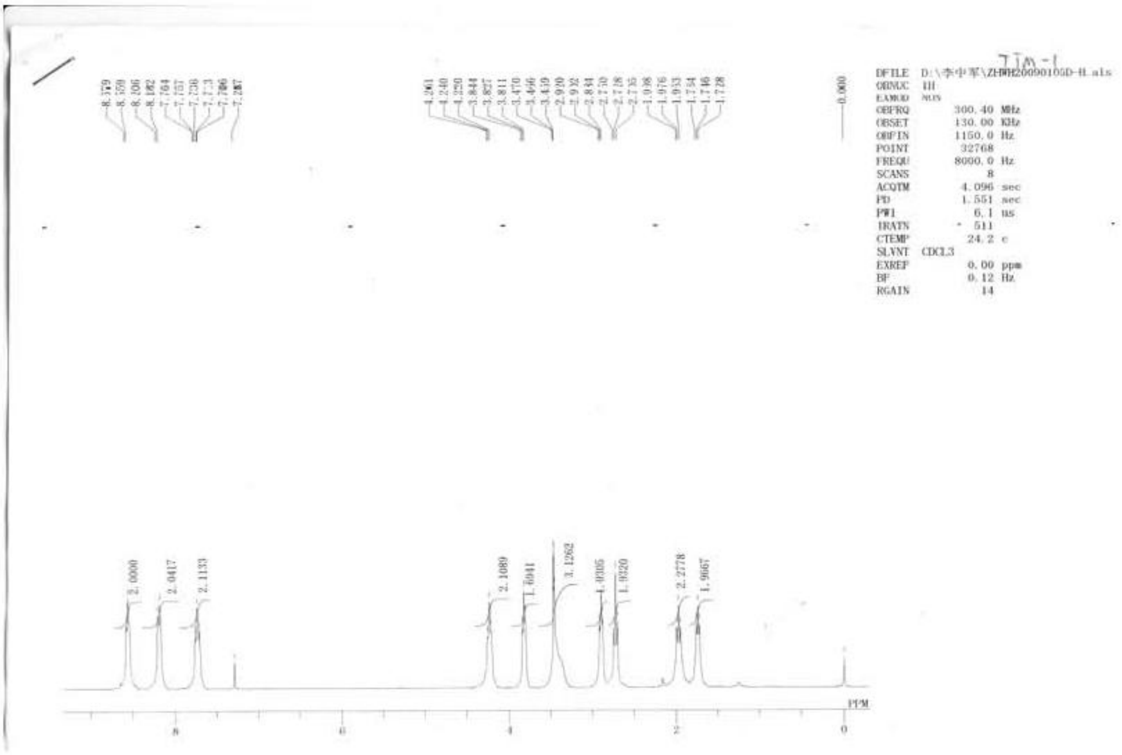

3b ^{13}C-NMR (75 MHz, $CDCl_3$).

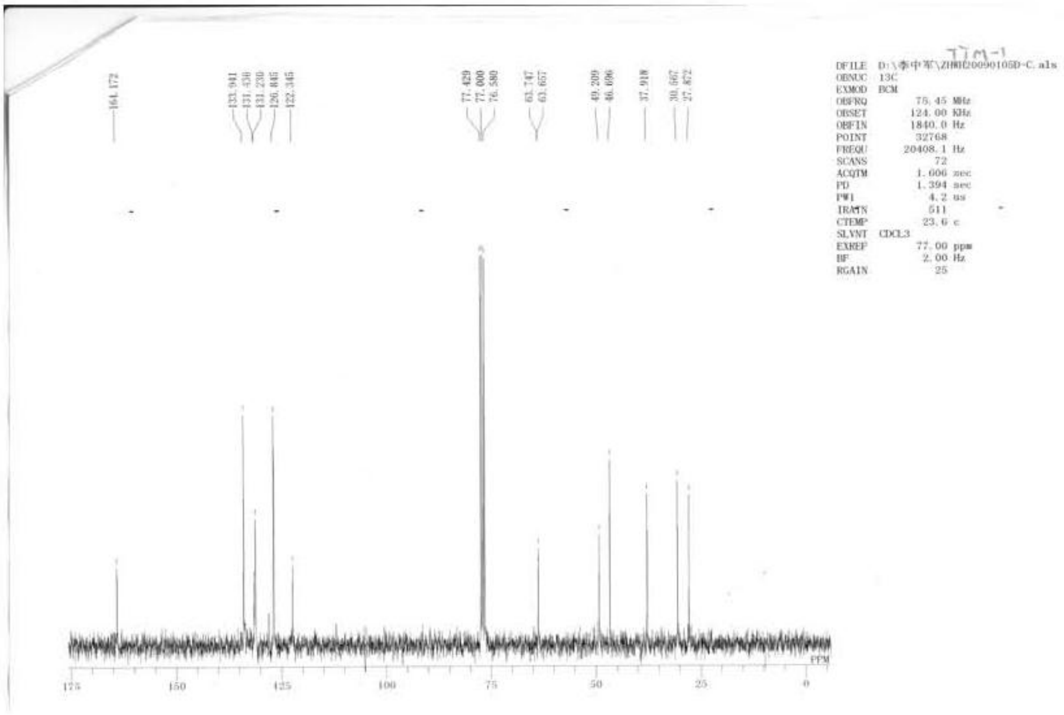

4b ^{1}H-NMR (300 MHz, $CDCl_3$).

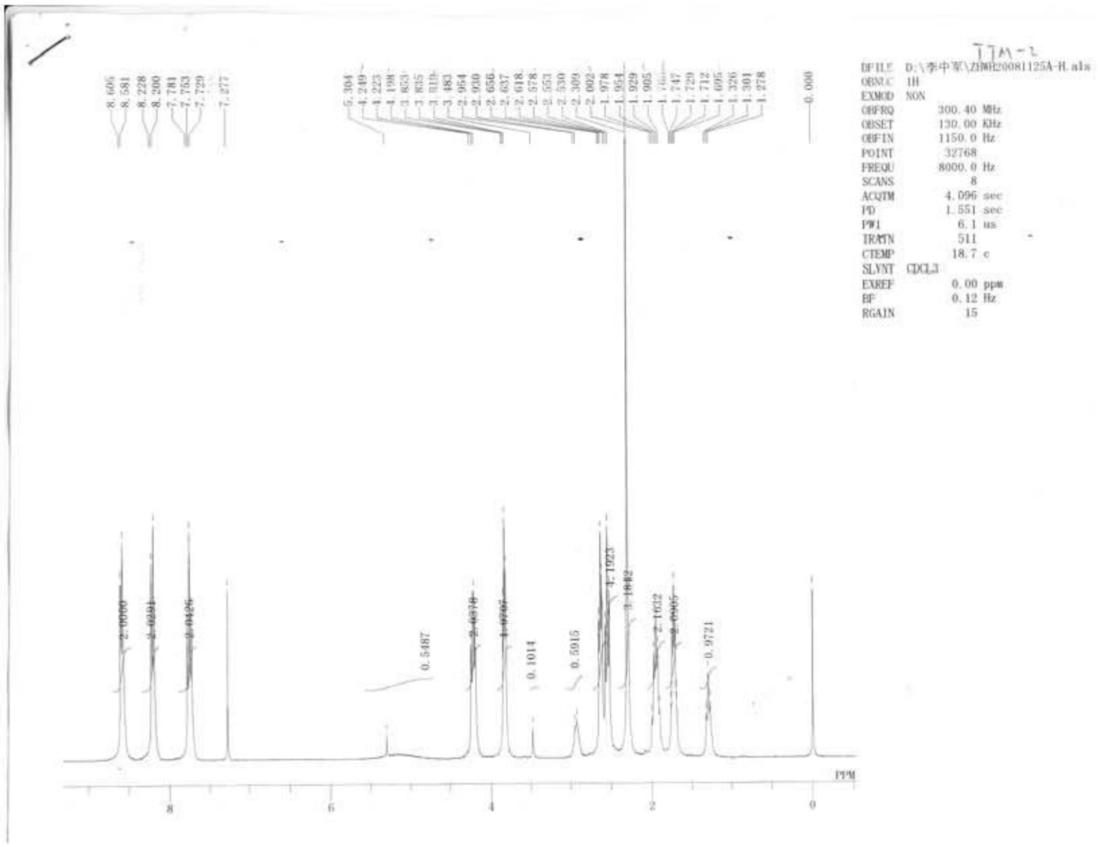

4b ^{13}C-NMR (75 MHz, $CDCl_3$).

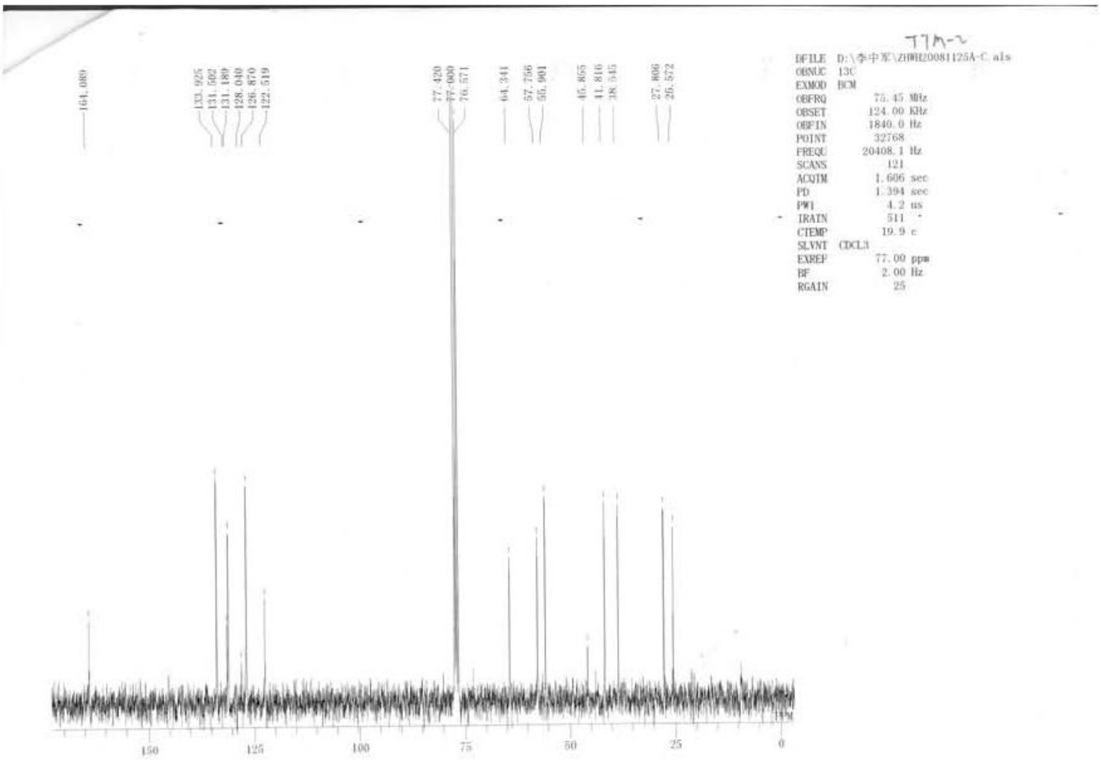

5a ^{1}H-NMR (300 MHz, $CDCl_3$).

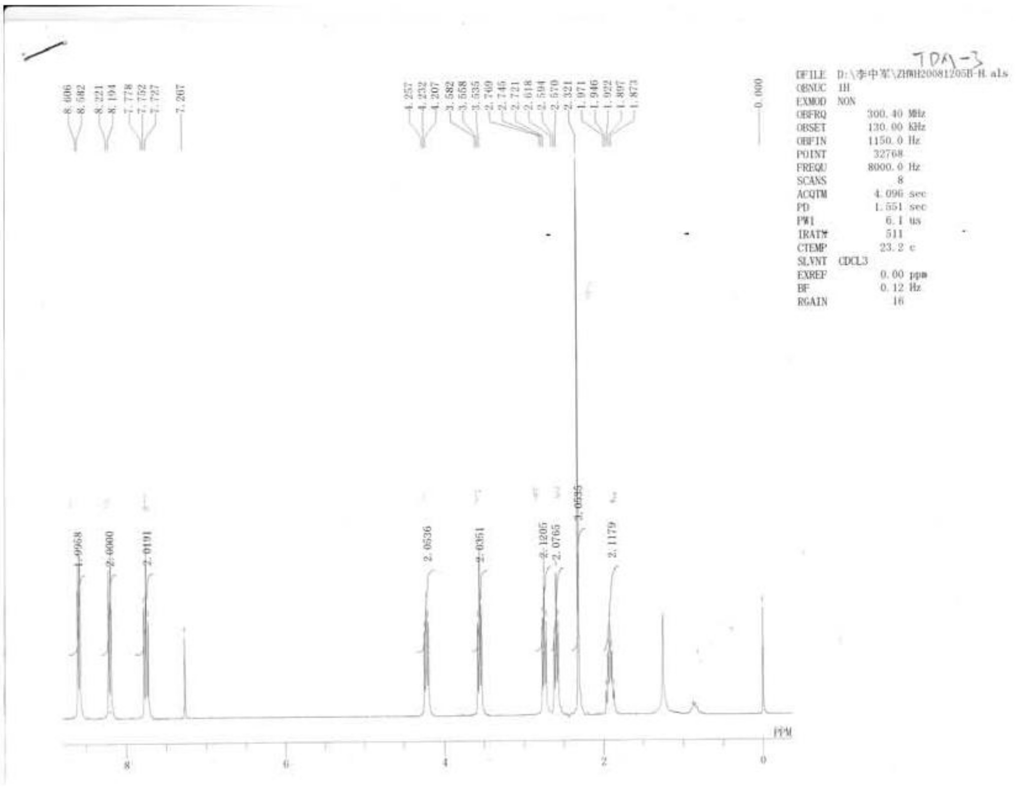

5a ^{13}C-NMR (75 MHz, $CDCl_3$).

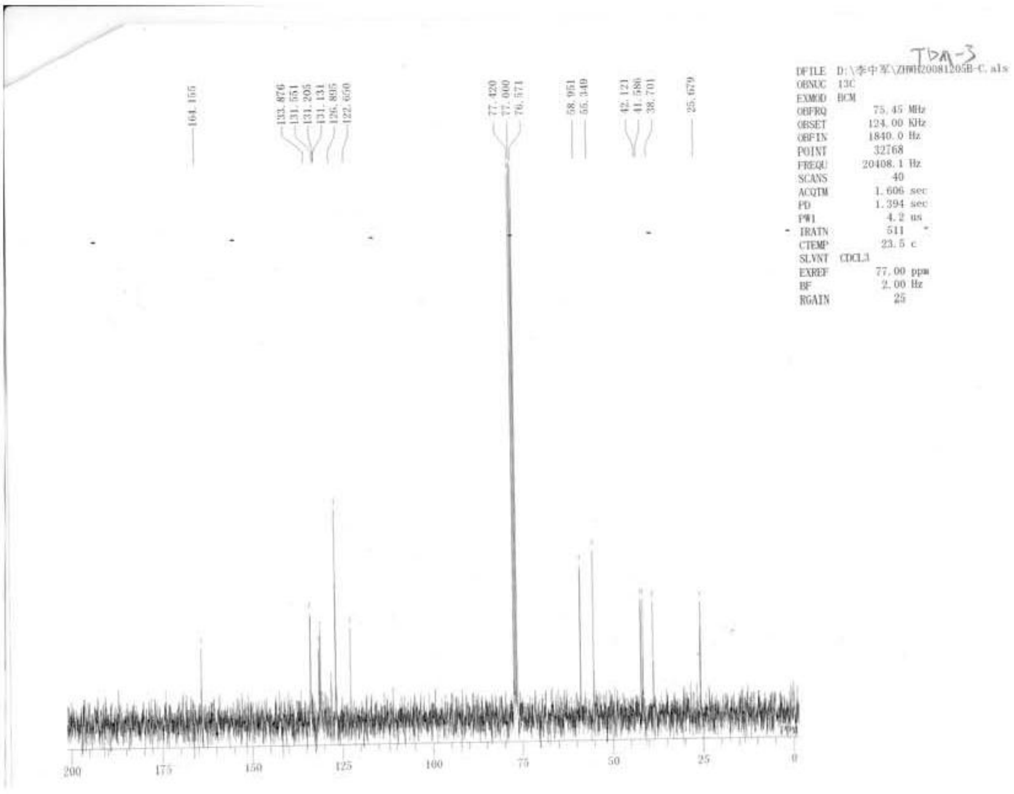

5b ^{1}H-NMR (300 MHz, $CDCl_3$).

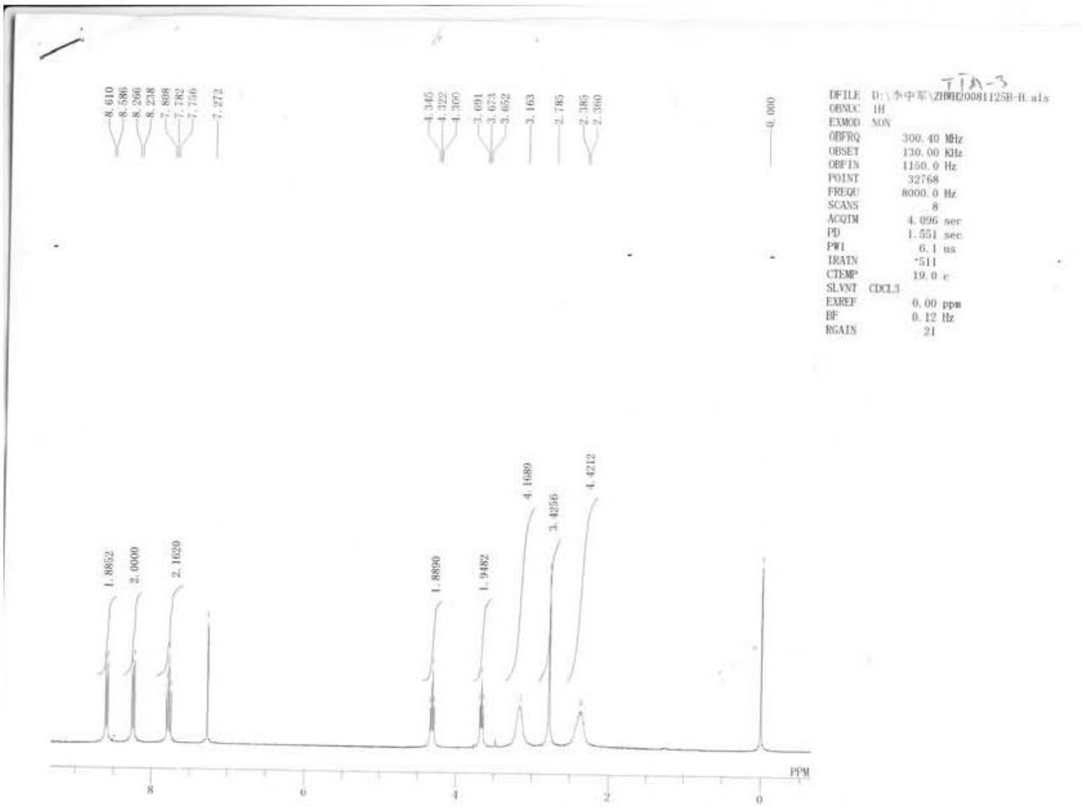

5b ^{13}C-NMR (75 MHz, $CDCl_3$).

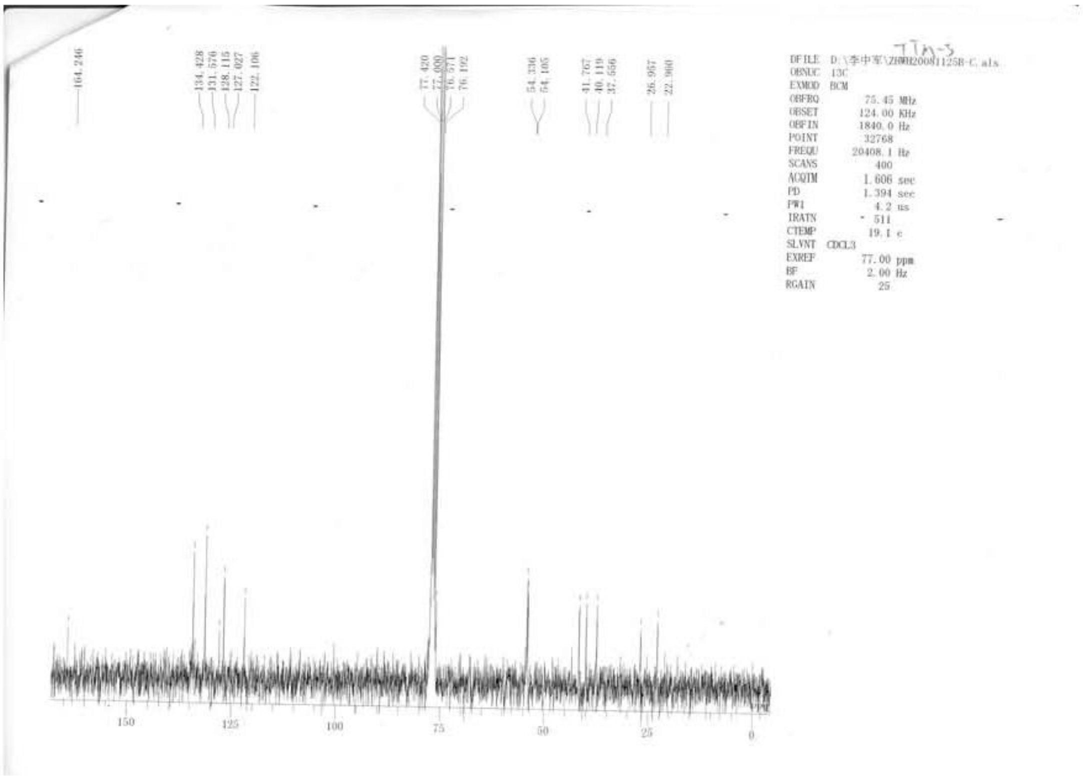

6a ^{1}H-NMR (300 MHz, $CDCl_3$).

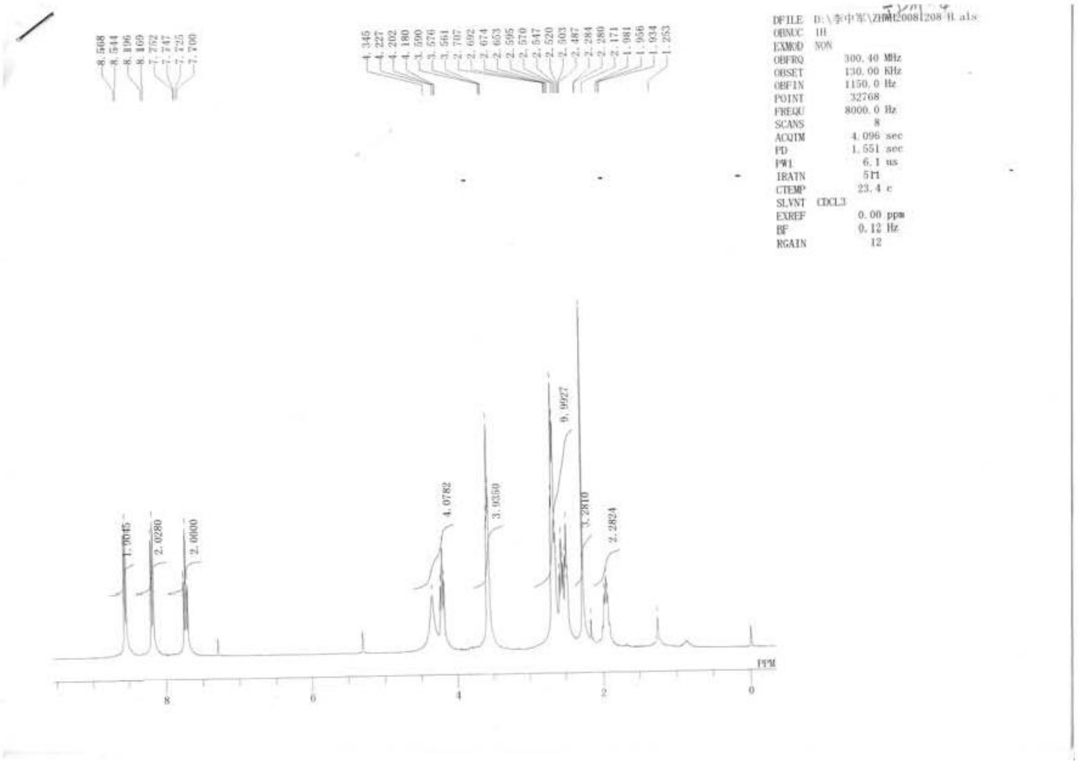

6a ^{13}C-NMR (75 MHz, $CDCl_3$).

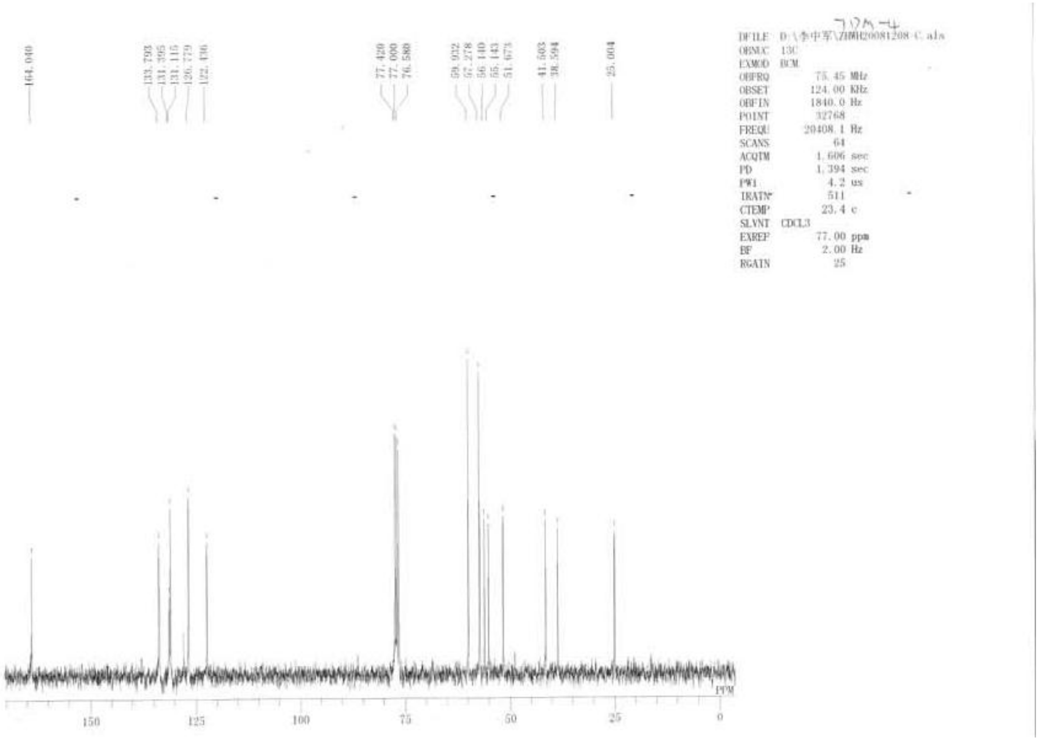

6b ^{1}H-NMR (300 MHz, $CDCl_3$).

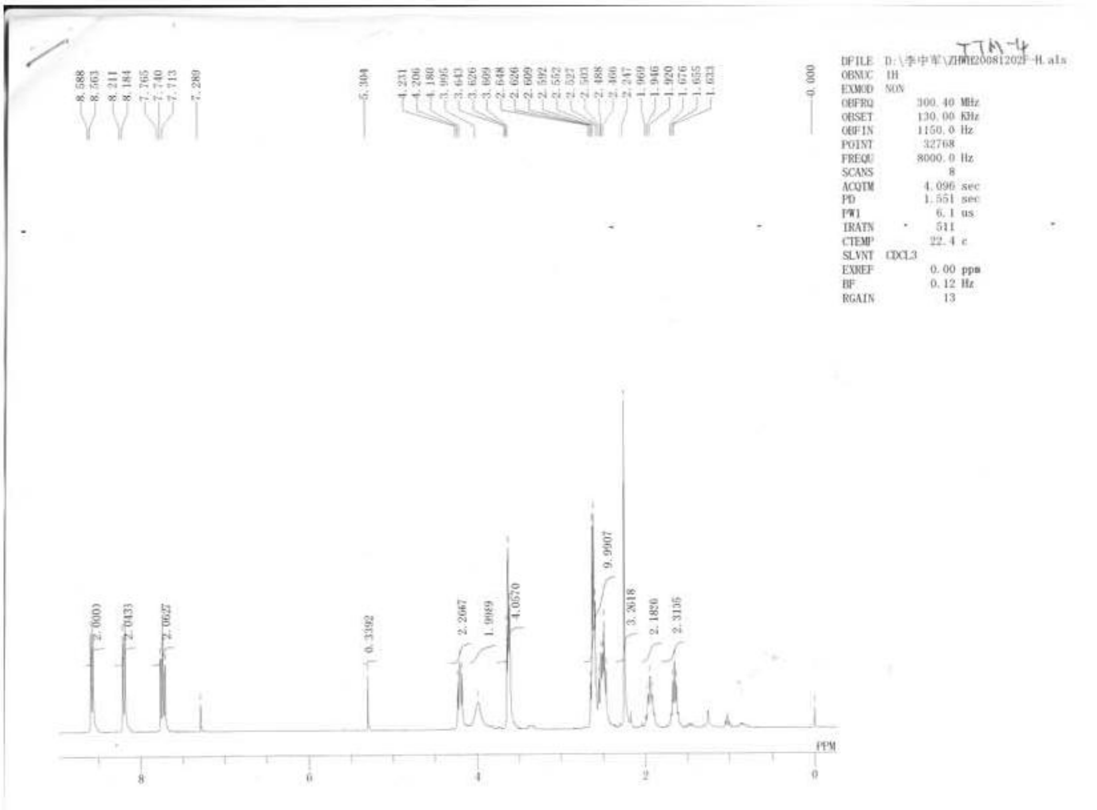

6b ^{13}C-NMR (75 MHz, $CDCl_3$).

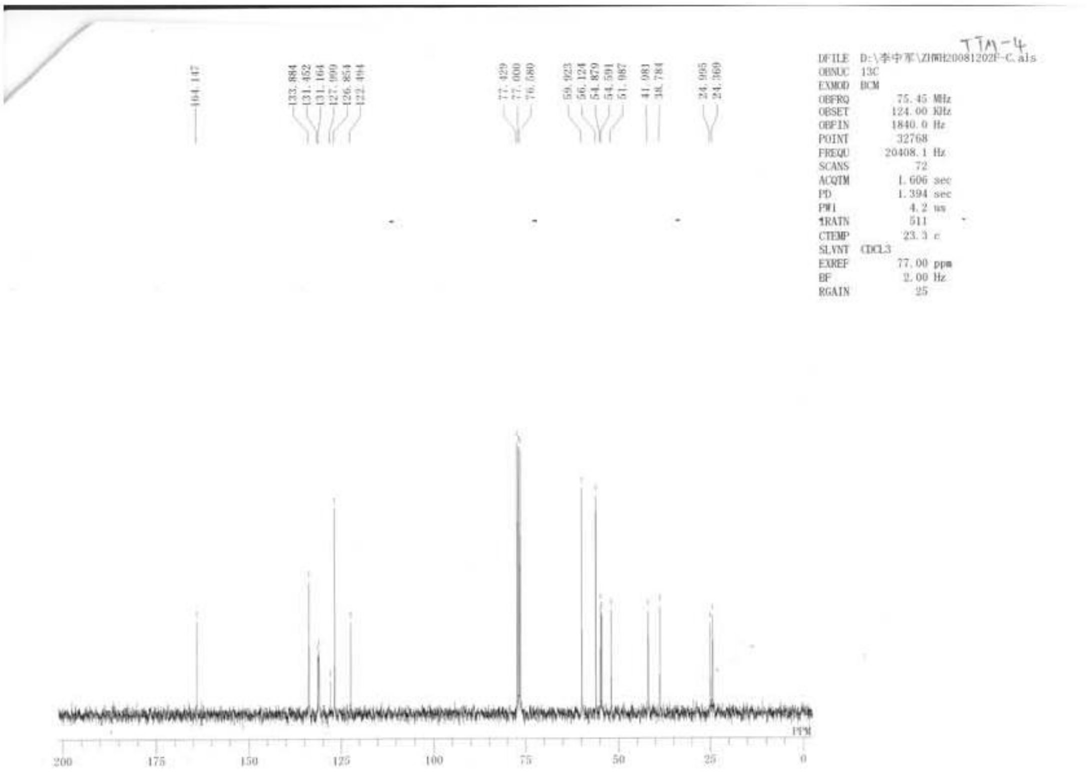

7a ^{1}H-NMR (300 MHz, $CDCl_3$).

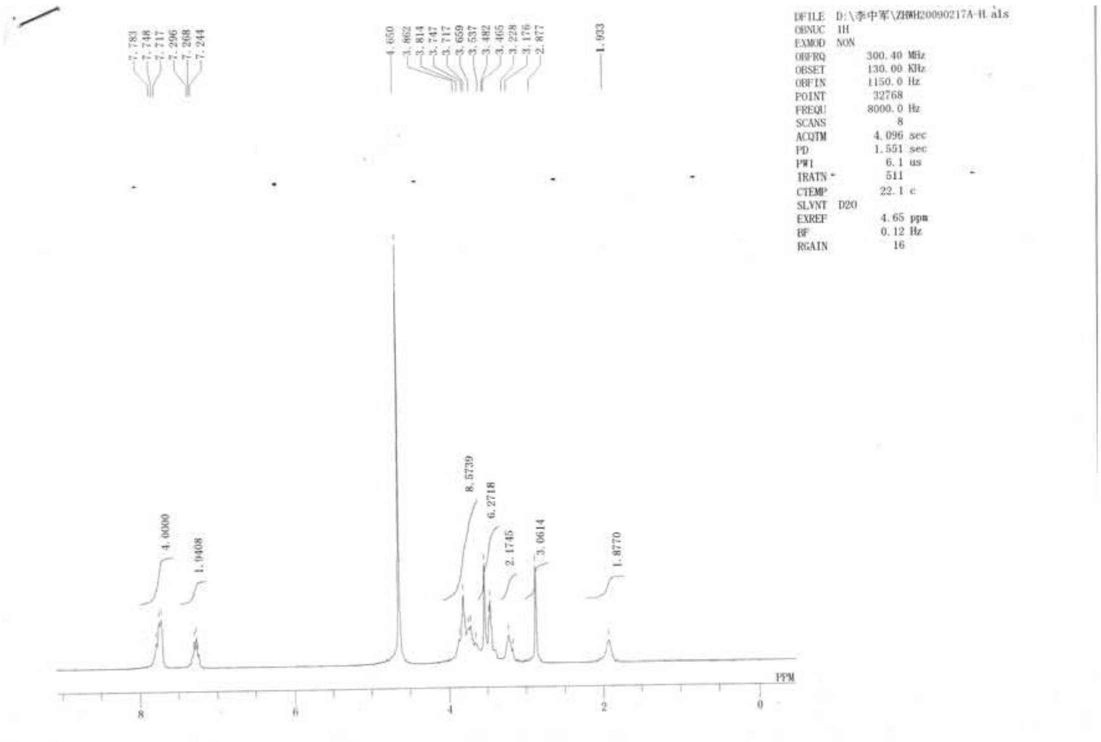

7a ^{13}C-NMR (75 MHz, $CDCl_3$).

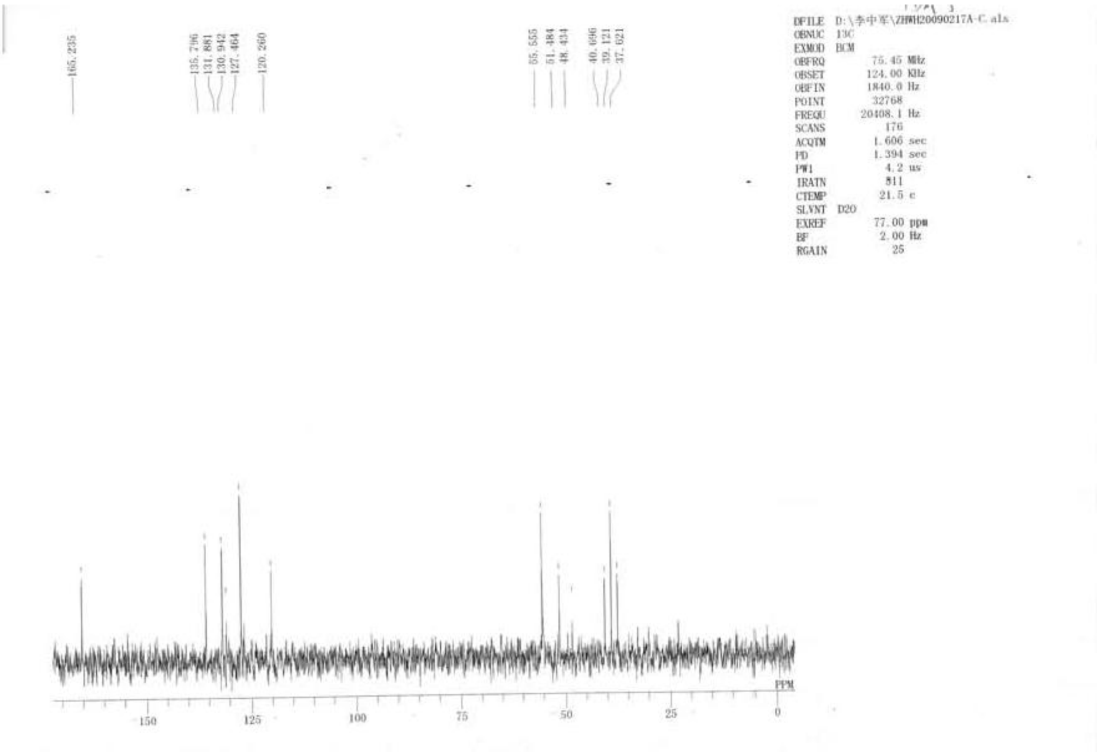

7b ^{1}H-NMR (300 MHz, $CDCl_3$).

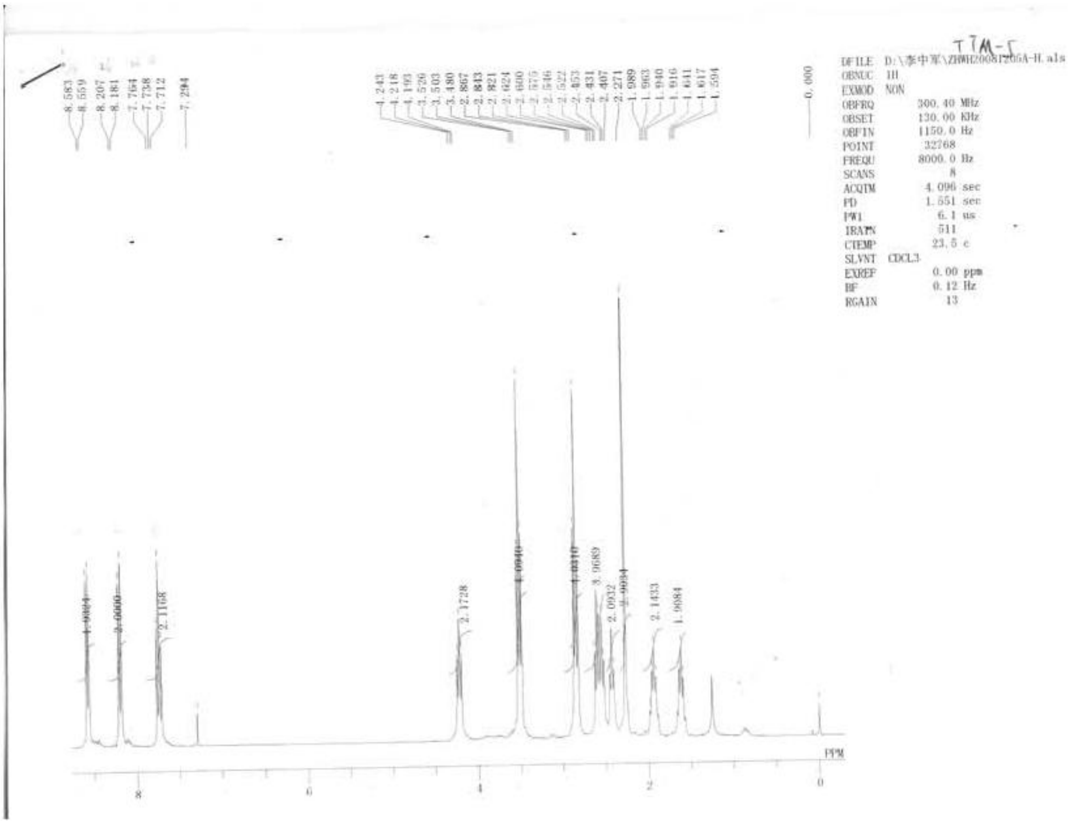

7b ^{13}C-NMR (75 MHz, $CDCl_3$).

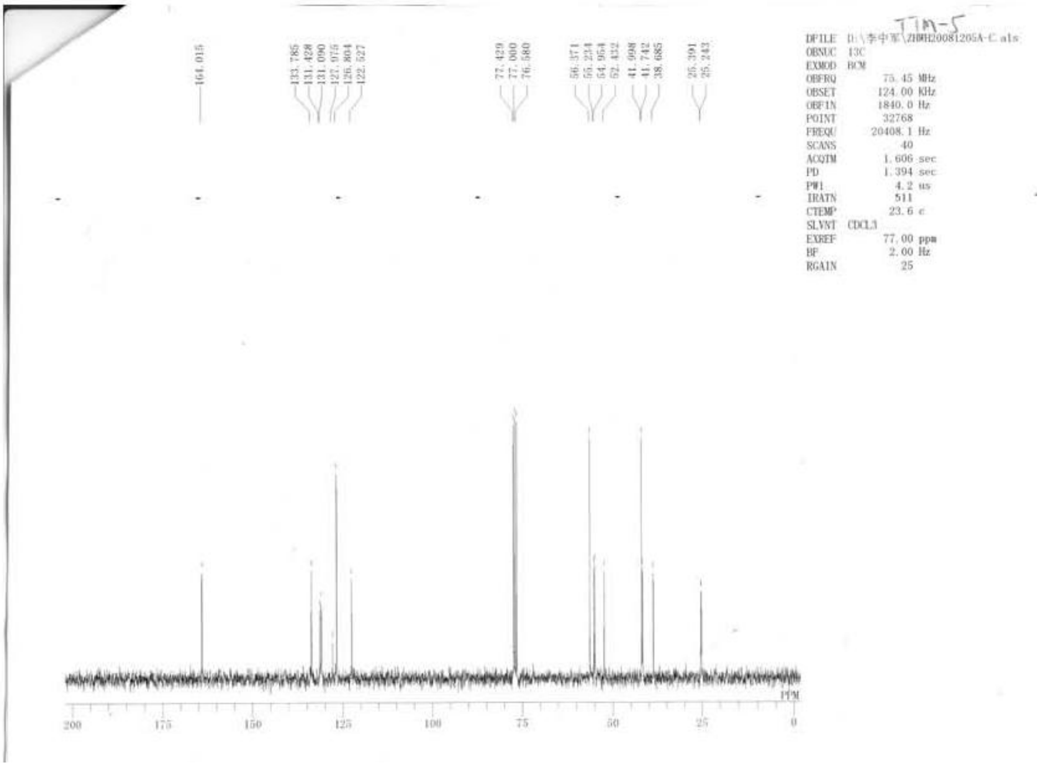

8a ^{1}H-NMR (300 MHz, $CDCl_3$).

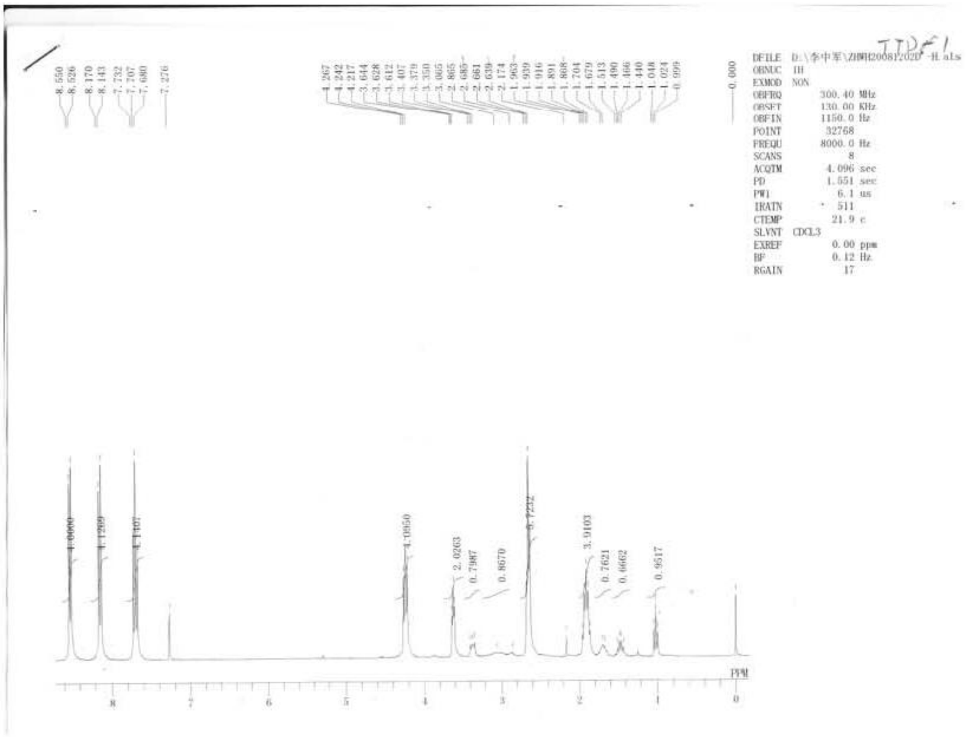

8a ^{13}C-NMR (75 MHz, $CDCl_3$).

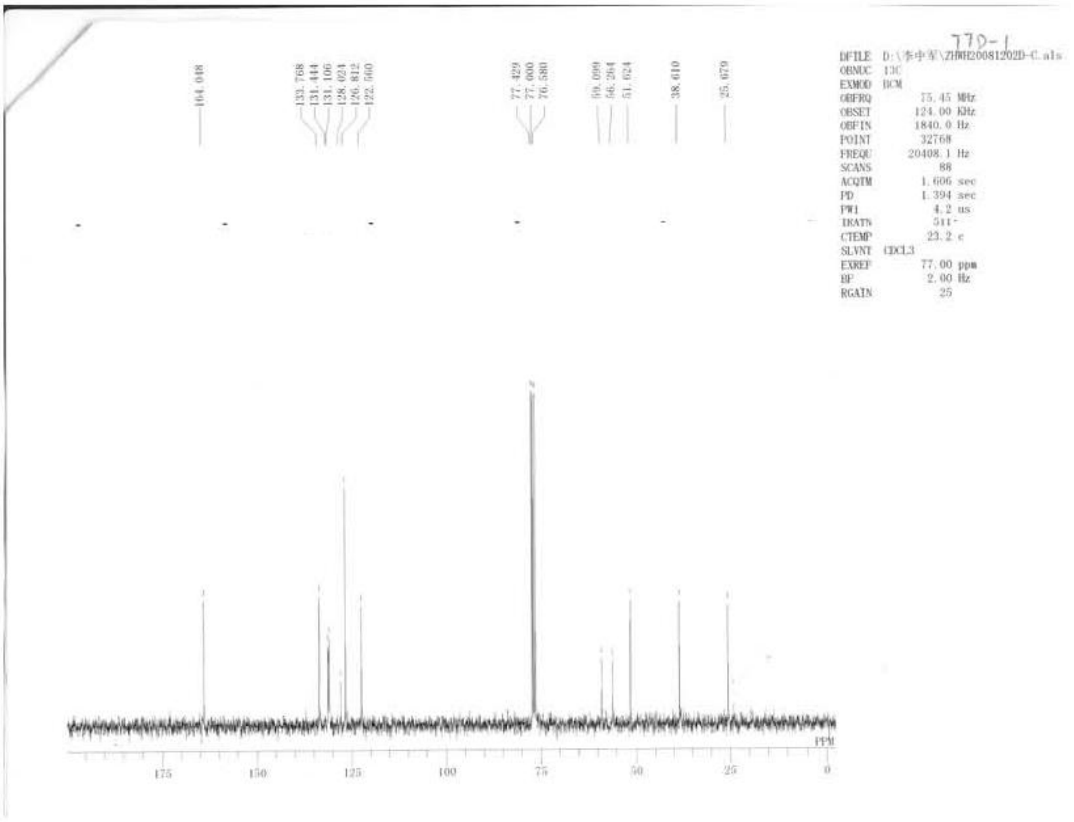

8b ^{1}H-NMR (300 MHz, $CDCl_3$).

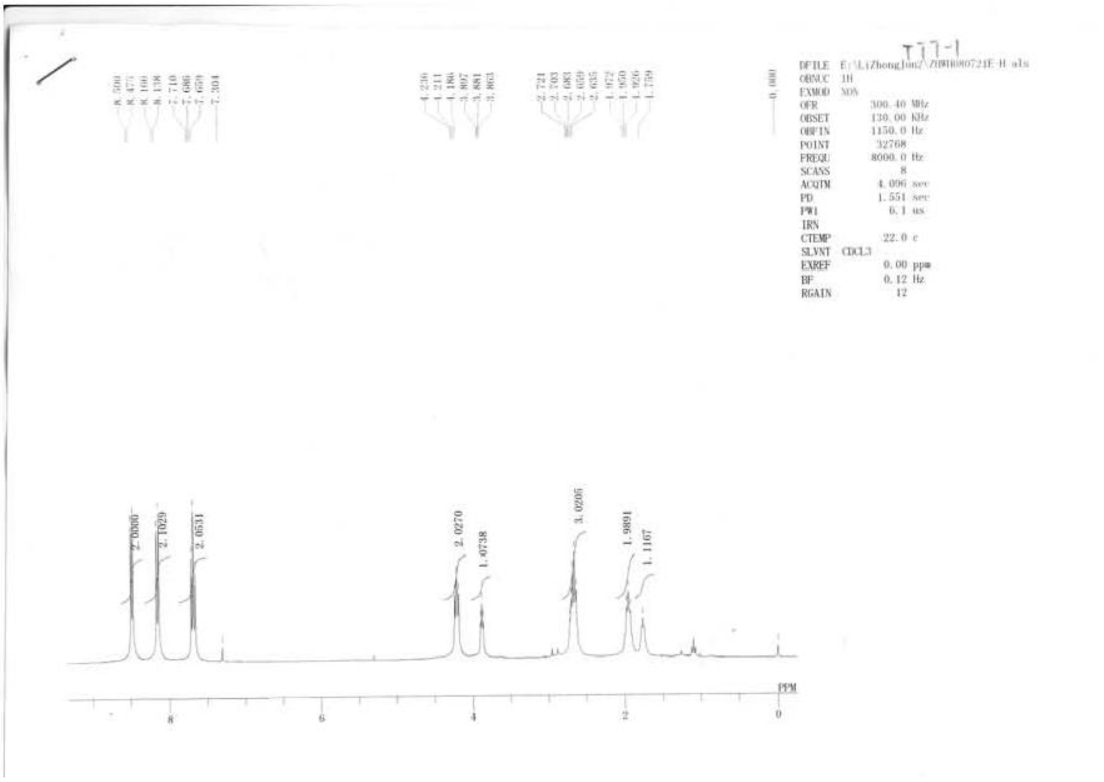

8b ^{13}C-NMR (75 MHz, $CDCl_3$).

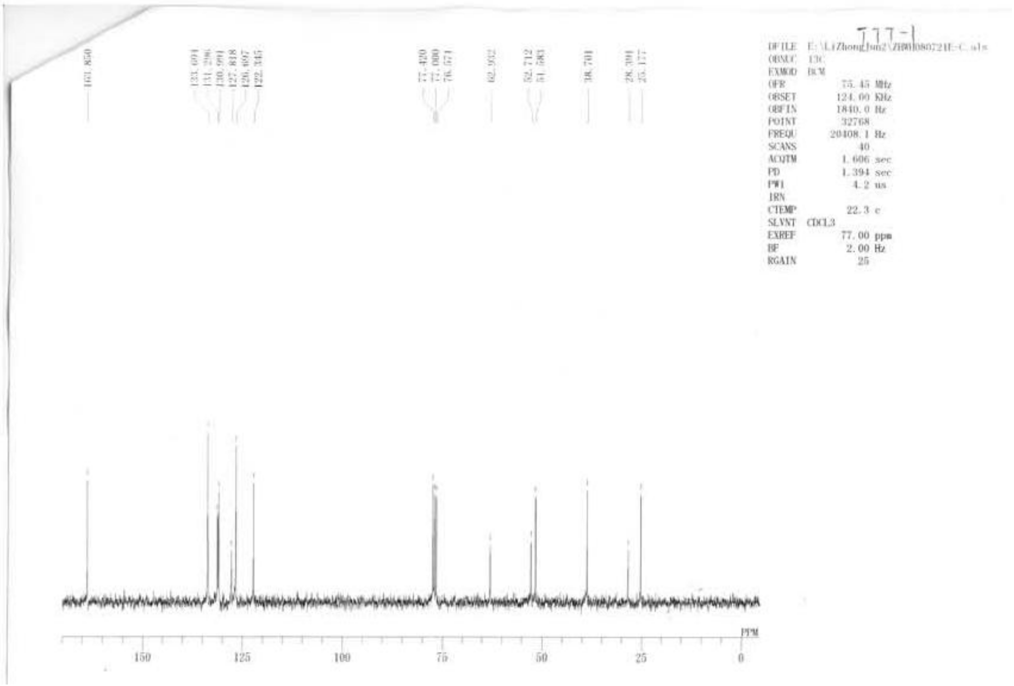

9b ^{1}H-NMR (300 MHz, $CDCl_3$).

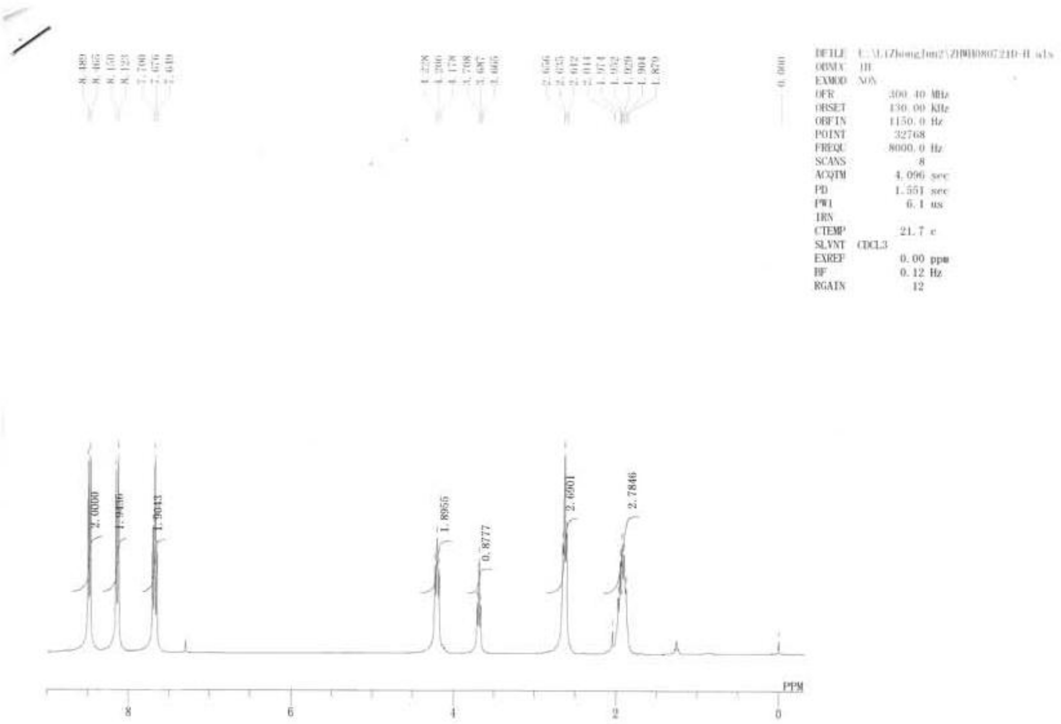

9b ^{13}C-NMR (75 MHz, $CDCl_3$).

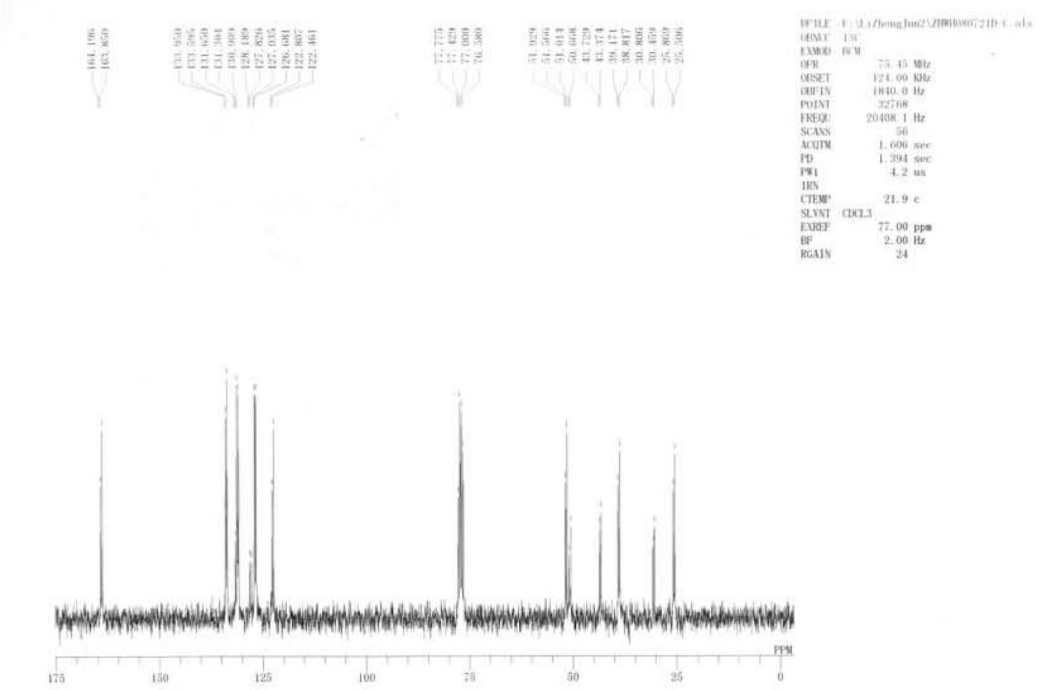

10a ^{1}H-NMR (300 MHz, $CDCl_3$).

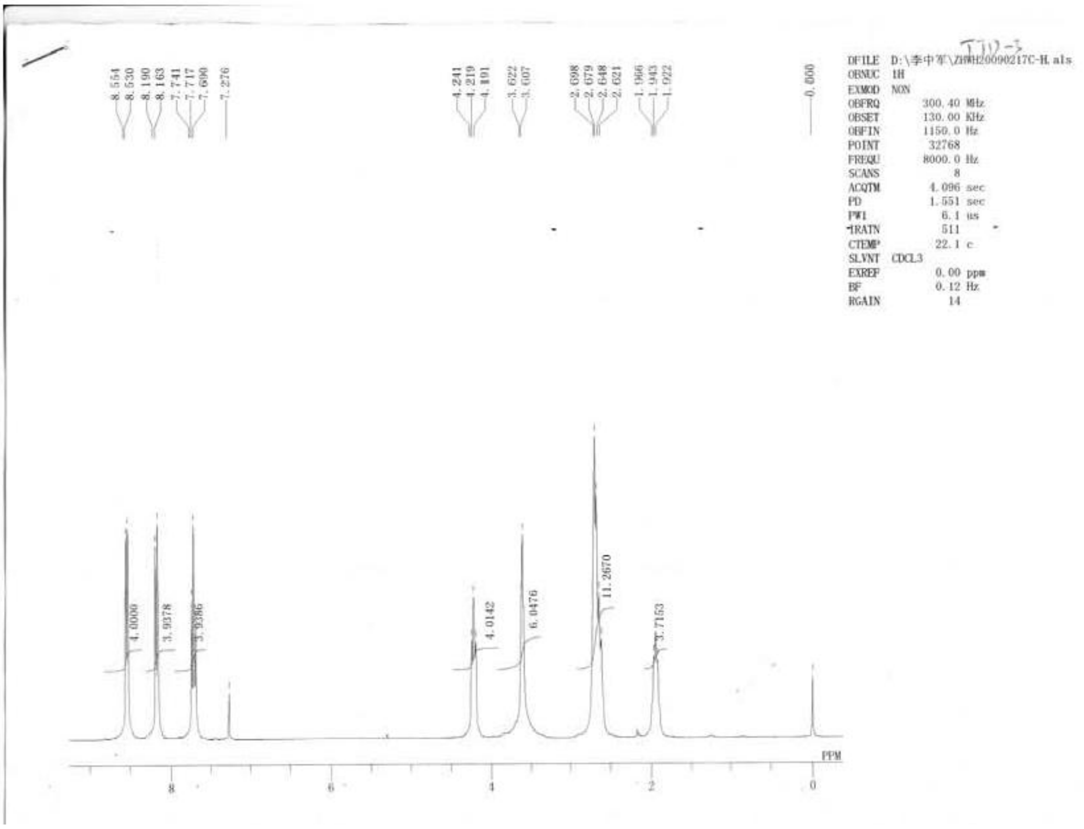

10a ^{13}C-NMR (75 MHz, $CDCl_3$).

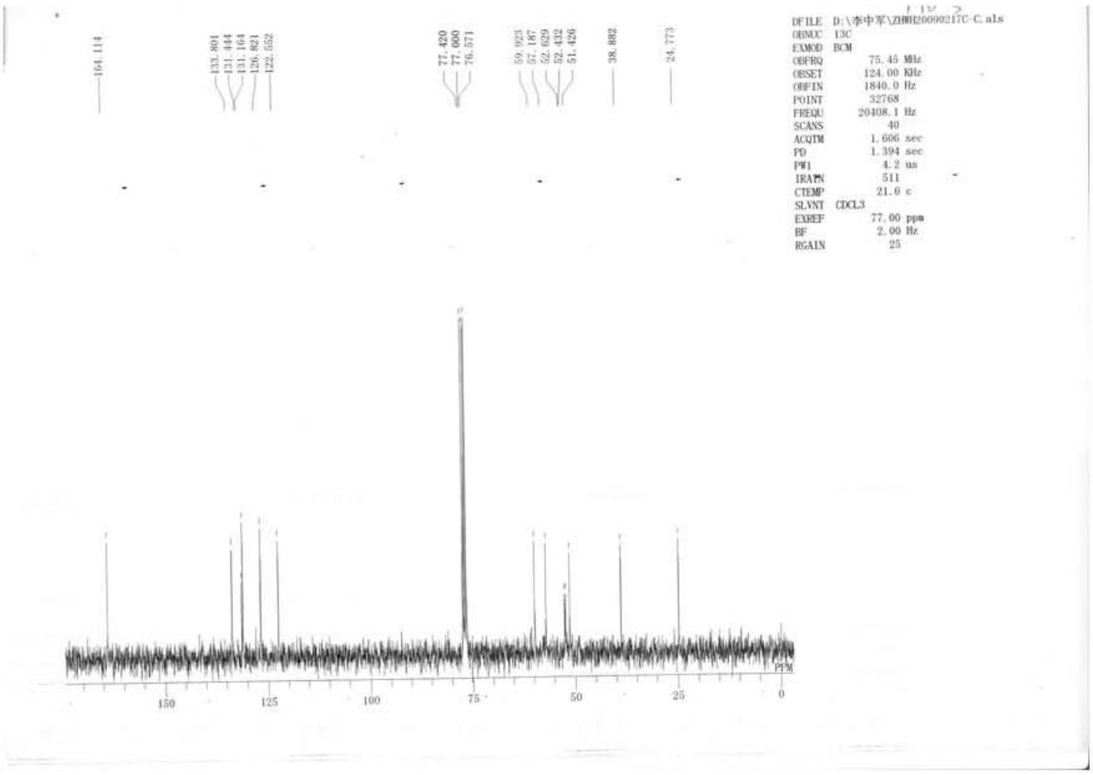

10b ^{1}H-NMR (300 MHz, $CDCl_3$).

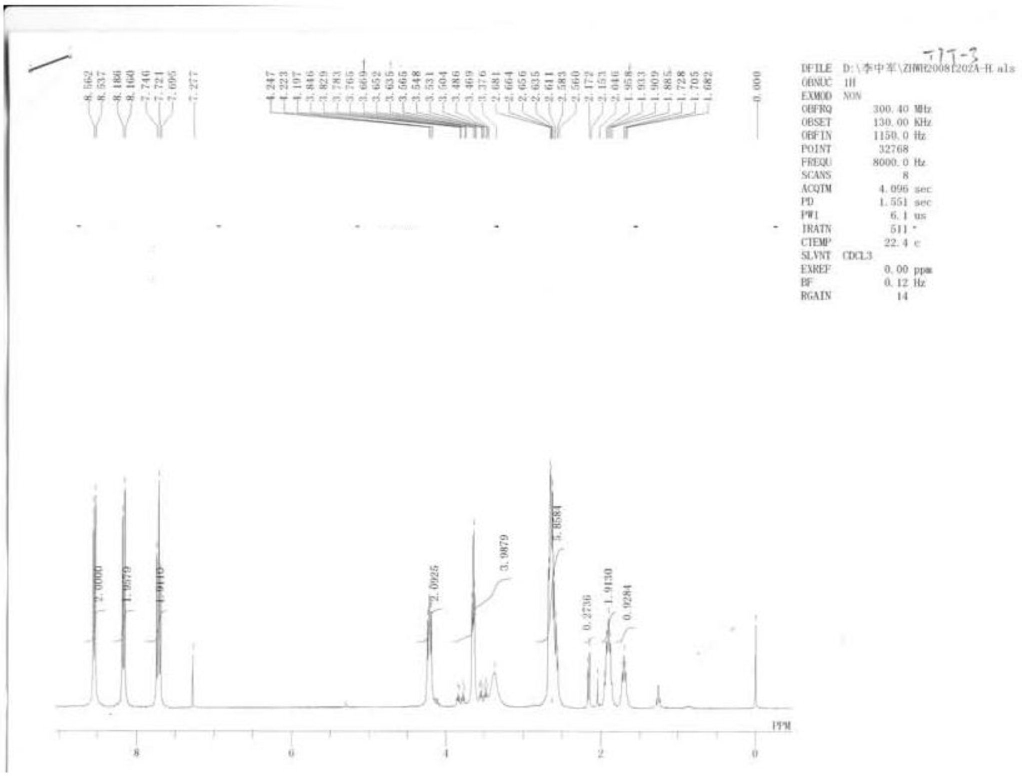

10b ^{13}C-NMR (75 MHz, $CDCl_3$).

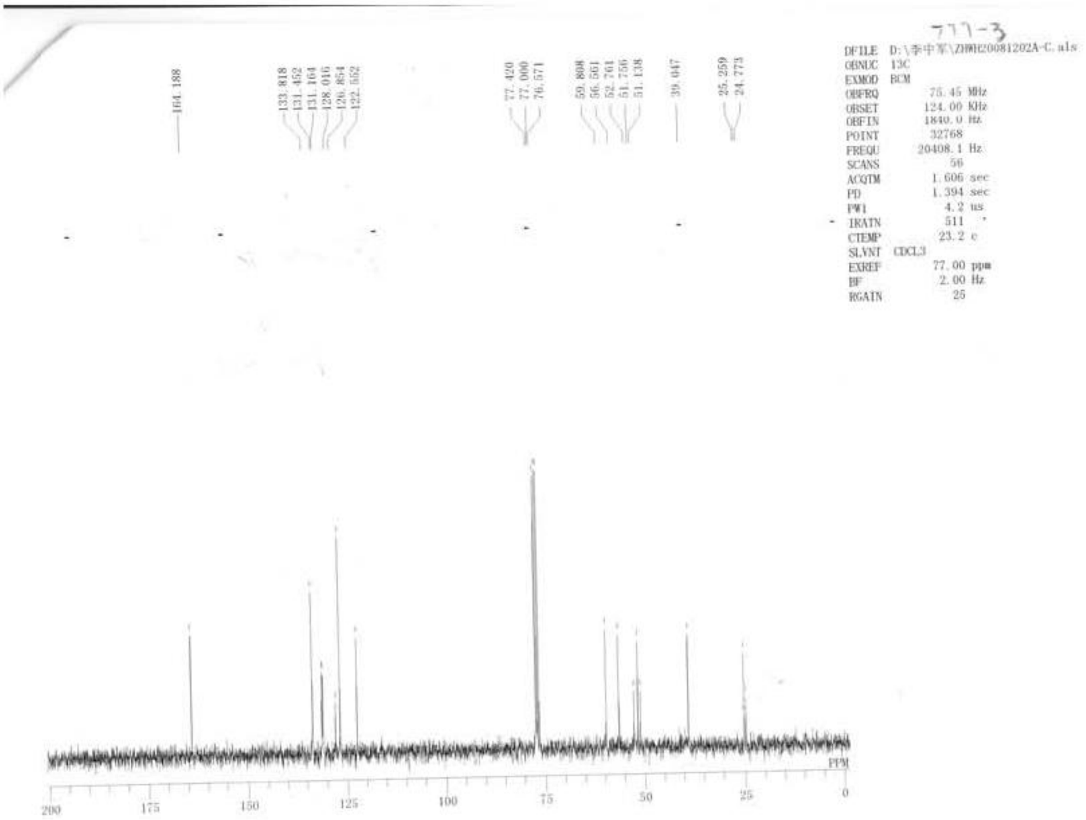

11a ^{1}H-NMR (300 MHz, $CDCl_3$).

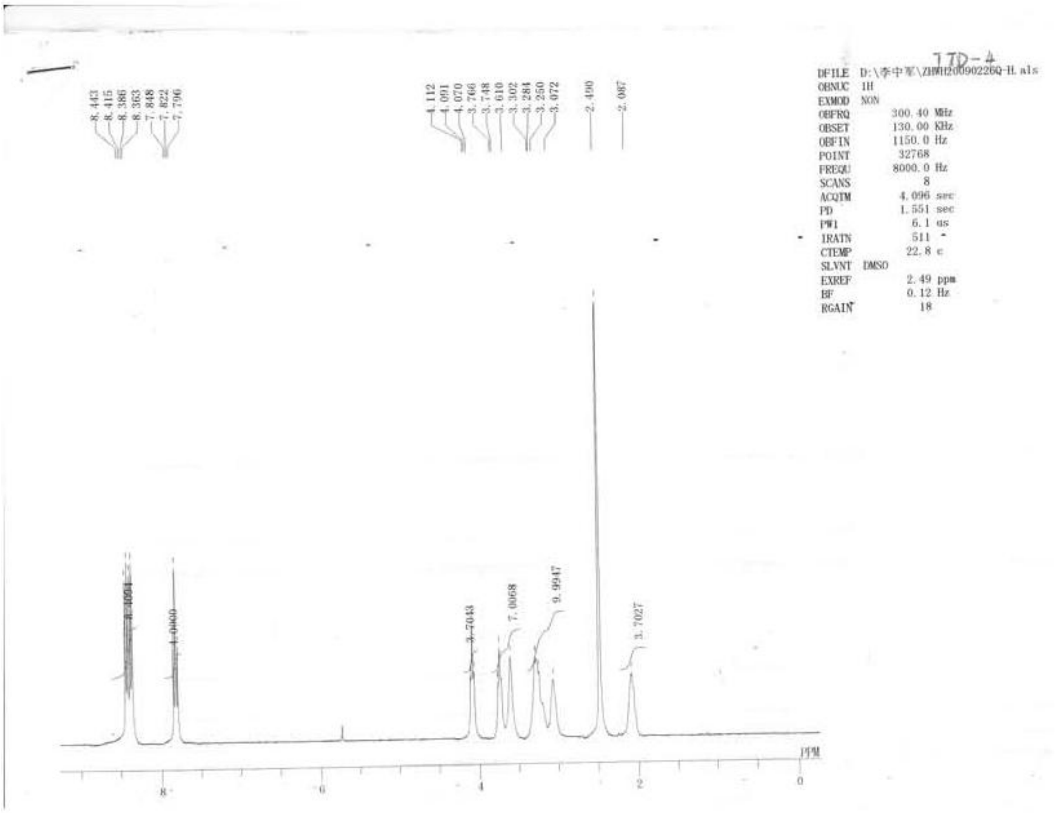

11a ^{13}C-NMR (75 MHz, $CDCl_3$).

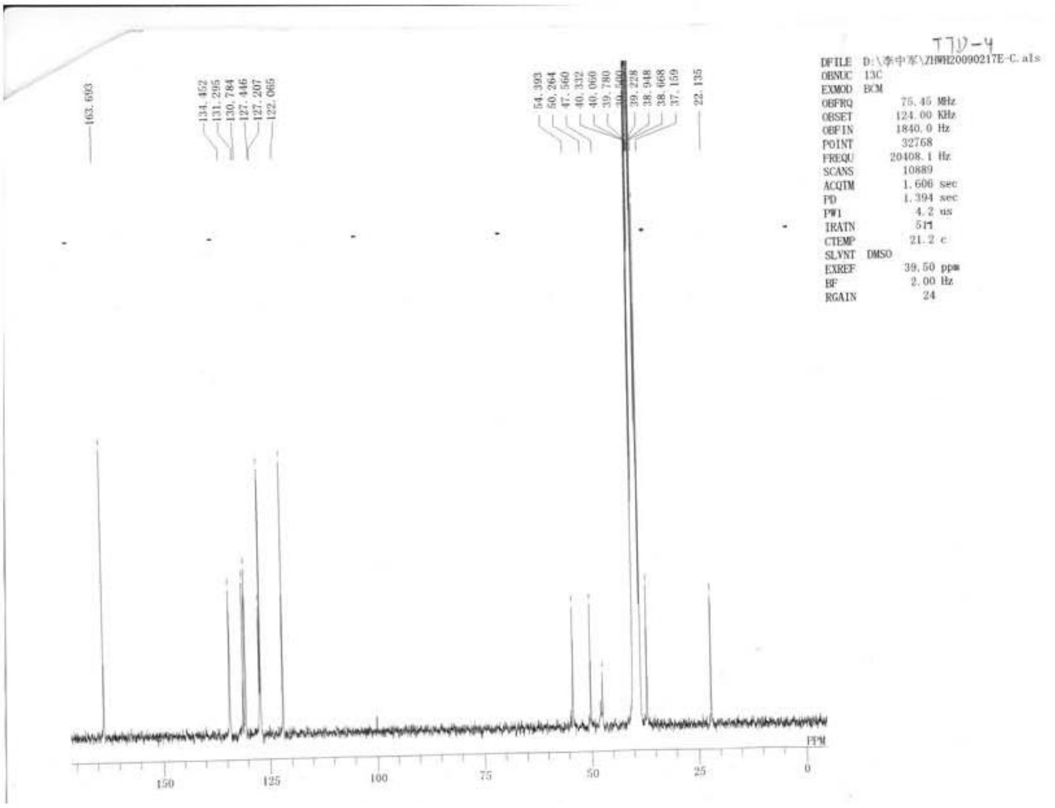

11b ^{1}H-NMR (300 MHz, $CDCl_3$).

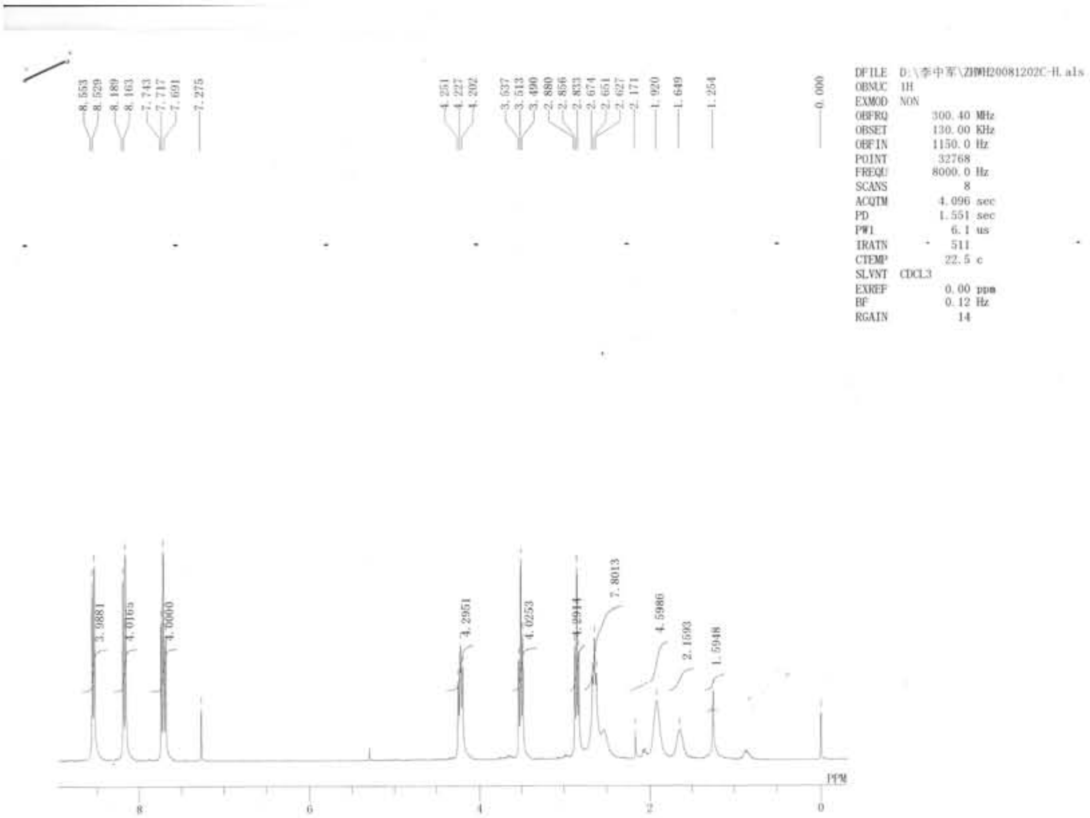

11b ^{13}C-NMR (75 MHz, $CDCl_3$).

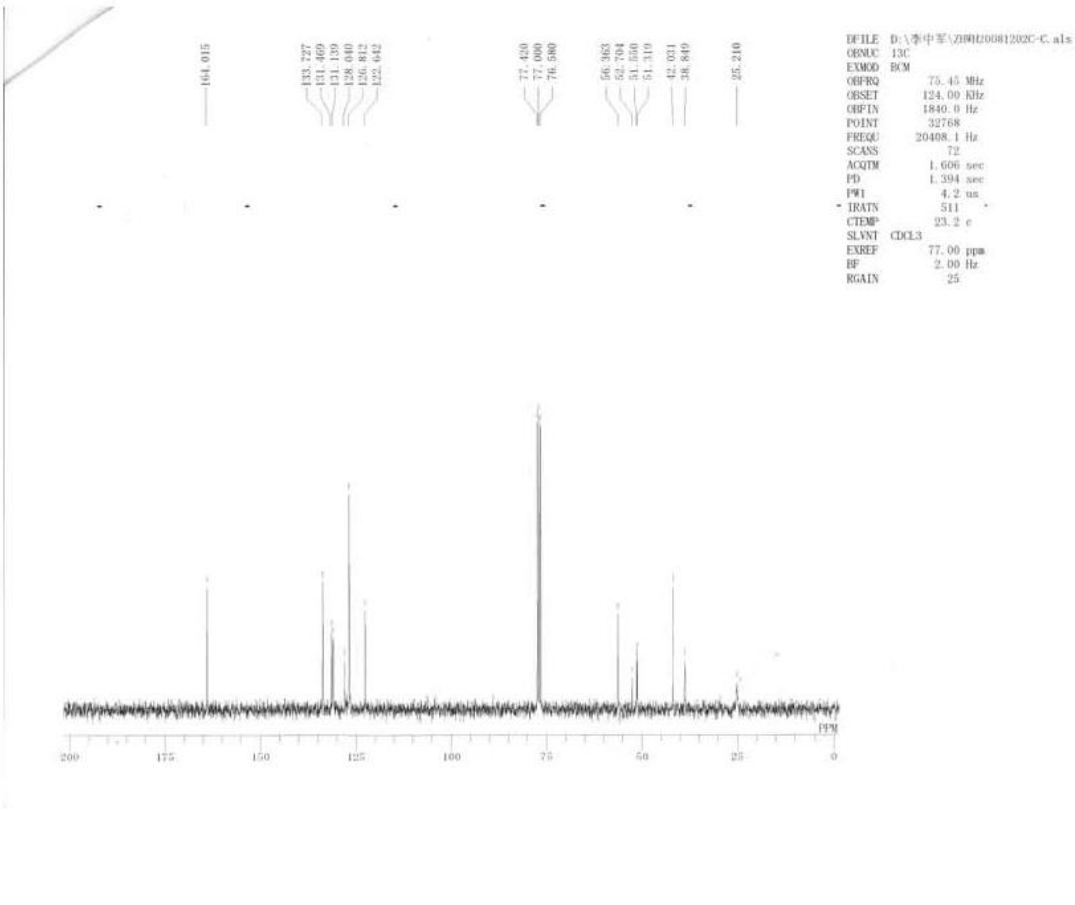

Supplement: Supplementary file 1 [file molecules-19-08803-s001.pdf]
